# Fatal police violence by race and state in the USA, 1980–2019: a network meta-regression

**DOI:** 10.1016/S0140-6736(21)01609-3

**Published:** 2021-10-02

**Authors:** GBD 2019 Police Violence US Subnational Collaborators

## Abstract

**Background:**

The burden of fatal police violence is an urgent public health crisis in the USA. Mounting evidence shows that deaths at the hands of the police disproportionately impact people of certain races and ethnicities, pointing to systemic racism in policing. Recent high-profile killings by police in the USA have prompted calls for more extensive and public data reporting on police violence. This study examines the presence and extent of under-reporting of police violence in US Government-run vital registration data, offers a method for correcting under-reporting in these datasets, and presents revised estimates of deaths due to police violence in the USA.

**Methods:**

We compared data from the USA National Vital Statistics System (NVSS) to three non-governmental, open-source databases on police violence: Fatal Encounters, Mapping Police Violence, and The Counted. We extracted and standardised the age, sex, US state of death registration, year of death, and race and ethnicity (non-Hispanic White, non-Hispanic Black, non-Hispanic of other races, and Hispanic of any race) of each decedent for all data sources and used a network meta-regression to quantify the rate of under-reporting within the NVSS. Using these rates to inform correction factors, we provide adjusted estimates of deaths due to police violence for all states, ages, sexes, and racial and ethnic groups from 1980 to 2019 across the USA.

**Findings:**

Across all races and states in the USA, we estimate 30 800 deaths (95% uncertainty interval [UI] 30 300–31 300) from police violence between 1980 and 2018; this represents 17 100 more deaths (16 600–17 600) than reported by the NVSS. Over this time period, the age-standardised mortality rate due to police violence was highest in non-Hispanic Black people (0·69 [95% UI 0·67–0·71] per 100 000), followed by Hispanic people of any race (0·35 [0·34–0·36]), non-Hispanic White people (0·20 [0·19–0·20]), and non-Hispanic people of other races (0·15 [0·14– 0·16]). This variation is further affected by the decedent's sex and shows large discrepancies between states. Between 1980 and 2018, the NVSS did not report 55·5% (54·8–56·2) of all deaths attributable to police violence. When aggregating all races, the age-standardised mortality rate due to police violence was 0·25 (0·24–0·26) per 100 000 in the 1980s and 0·34 (0·34–0·35) per 100 000 in the 2010s, an increase of 38·4% (32·4–45·1) over the period of study.

**Interpretation:**

We found that more than half of all deaths due to police violence that we estimated in the USA from 1980 to 2018 were unreported in the NVSS. Compounding this, we found substantial differences in the age-standardised mortality rate due to police violence over time and by racial and ethnic groups within the USA. Proven public health intervention strategies are needed to address these systematic biases. State-level estimates allow for appropriate targeting of these strategies to address police violence and improve its reporting.

**Funding:**

Bill & Melinda Gates Foundation, National Institute on Minority Health and Health Disparities, and National Heart, Lung, and Blood Institute.

## Introduction

The Global Burden of Diseases, Injuries, and Risk Factors Study (GBD) in 2019 found that police conflict and executions accounted for 293 000 global deaths (95% uncertainty interval [UI] 215 000–344 000) from 1980 to 2019.^1^ In 2019, the USA accounted for 13·2% (95% UI 11·6–15·1) of the 8770 global deaths (7710–9930) due to police conflict while only accounting for 4% of the global population;^1^ police conflict and executions was the estimated cause of death for 1150 deaths (998–1310) in the USA.^1^ The burden of police violence fatalities in the USA is known to fall disproportionately on Black, Indigenous, and Hispanic populations.^2^, ^3^, ^4^, ^5^ Recent studies suggest that over the life course, about one in every 1000 Black men are killed by the police in the USA, making them 2·5 times more likely to be killed by police than White men.^2^ Black women are about 1·4 times more likely to be killed by police than are White women.^2^ Systemic and direct racism, manifested in laws and policies as well as personal implicit biases, result in Black, Indigenous, and Hispanic Americans being the targets of police violence.^6^, ^7^, ^8^, ^9^

Within GBD, deaths due to police conflict and executions include civilians killed by police, police killed by civilians, and government-led executions.^1^ Police violence is defined in GBD as police-related altercations leading to death or bodily harm. For the purpose of this study, we estimate numbers of civilians killed by police disaggregated by race and ethnicity, referred to throughout this work as fatal police violence.

**Research in context**

**Evidence before this study**

Current data on deaths from police violence are constrained by the limitations of government-run vital registration systems. Vital registration data are often considered high quality for cause of death estimation; however, vital registration systems can be biased. Considerable evidence in the USA suggests government vital registration data under-report police violence. We completed a systematic review of databases on police violence in the USA by searching the terms “police violence OR killing OR shooting OR conflict database” on June 2, 2020, using Google and Google Scholar.

**Added value of this study**

We evaluated the extent of under-reporting of deaths due to police violence in the USA at the state level by race and ethnicity by comparing vital registration data to three non-governmental, open-source databases: Fatal Encounters, Mapping Police Violence, and The Counted. We chose these databases on the basis of the following factors: (1) their comprehensive coverage of the entire USA, (2) their detailed inclusion of both the state of death and race or ethnicity of decedents, and (3) their consideration of all forms of violence. To correct for under-reporting in US vital registration data, we developed a statistical framework using both open-source and government data sources to provide appropriately revised estimates of deaths due to police violence stratified by age, sex, year of death, and race and ethnicity for each state within the USA from 1980 to 2019. The revised estimates highlight the extent to which these deaths are under-reported and the disproportionate effect of police violence on non-Hispanic Black, non-Hispanic Indigenous, and Hispanic Americans.

**Implications of all the available evidence**

Stark inequities in the burden of police killings by race and ethnicity within the USA highlight the urgent need to address systemic racism within the US police force. The ability to accurately compare rates of deaths between countries is pivotal in addressing systematic issues across global policing systems. This study can serve as a framework in guiding future research to address the under-reporting of police violence in additional countries. Future work remains for researchers and public health officials to swiftly adopt open-source data-collection initiatives to provide accurate estimates and advocate for policy change to address this long-neglected public health crisis.

The GBD 2019 estimates for police conflict and executions were corrected for under-reporting using a similar method as described in this Article.^1^ These corrections will also be applied in GBD 2020 and in future iterations of GBD. Before GBD 2019, the exclusive data source used for fatal police violence in GBD was the National Vital Statistics System (NVSS), leading to underestimation of fatal police violence by 52·6–67·8% each year from 1980 to 2017.

Movements against racially motivated violence have been long standing throughout the USA's history. Recent tragedies have spurred social movements such as Black Lives Matter (initiated in 2013 by community organisers Patrisse Cullors, Alicia Garza, and Opal Tometi in response to the killing of Trayvon Martin)^10^ and public declarations identifying police violence and racism as a public health crisis.^11^, ^12^ Violence is a public health issue, affecting both physical and mental health and undermining individual and community safety and wellbeing.^13^, ^14^, ^15^ Since the creation of Black Lives Matter, the crisis of police violence and its associated systemic racism has been broadly identified as a public health concern in other high-income nations besides the USA,^16^ and the international spotlight on racial disparities in US police violence has incited protests globally.^17^

Despite the magnitude of the loss of life and the evident disproportionate burden of deaths from the police on Black, Indigenous, and Hispanic people in the USA, these deaths can be misclassified and subsequently undercounted in official statistics.^18^, ^19^, ^20^, ^21^ Many countries, including European and Latin American countries, Australia, and high-income Asian nations, rely on government-run vital registration systems to collect cause of death data.^22^, ^23^, ^24^, ^25^, ^26^, ^27^, ^28^, ^29^ Although vital registration systems in many countries are considered to be reliable sources on causes of death,^1^, ^30^, ^31^, ^32^ they present a potential conflict of interest for deaths from police violence, since the same state responsible for violence is also responsible for reporting it.^3^, ^18^ The NVSS is a government system coordinated by the National Center for Health Statistics to provide guidance, collate, and standardise vital registration data collected by US states. The NVSS collates all death certificates issued in the USA, including each decedent's age, sex, race, Hispanic ethnicity, place of residence, date of death, and cause of death.^33^, ^34^ Physicians are typically responsible for filling out the cause of death section of the death certificate; however, a medical examiner or coroner who may or may not also be a physician will do so for homicides or cases where there is suspicion of crime or foul play, including police violence.^19^, ^35^ Systemic misclassification and undercounting of deaths due to police violence in USA vital registration data has been well documented.^18^, ^19^, ^20^, ^21^

To address the public health crisis of police violence, reform is needed on how to document and respond to such violence. We have devised a framework to quantify and adjust for under-reporting in the NVSS using open-source data in the USA. In this study, we compared deaths identified as police violence in the NVSS to three outside, non-governmental databases that rely on open-source information: Fatal Encounters, Mapping Police Violence, and The Counted. The primary purpose of this Article is to examine and resolve the impact of under-reporting in the NVSS on the estimation of police violence in the USA at the state, race, and ethnicity level by developing a method to correct for under-reporting in these data. This method has the potential to provide a framework for other countries with similar vital registrations systems to correct their police violence data and to encourage the use of open-source data collection, allowing for global comparison of this key cause of death. The manuscript was produced as part of the GBD Collaborator Network and in accordance with the GBD Protocol.^36^

## Methods

### Overview

This analysis complied with the Guidelines for Accurate and Transparent Health Estimates Reporting (GATHER).^37^ The full GATHER checklist is provided in the appendix (p 13). This study is a subanalysis of GBD, which the University of Washington Institutional Review Board has approved under IRB ID 9060. A flowchart of our methods for the estimation of police violence can be found in the appendix (p 20).

### Data seeking

To find databases that capture fatal police violence more accurately than the NVSS, we searched the terms “police violence OR killing OR shooting OR conflict database” on June 2, 2020, using Google and Google Scholar. We included in our analyses all databases referenced in the search results that met the following criteria: (1) inclusion of both firearm-related and non-firearm-related deaths, (2) inclusion of state and race or ethnicity detail, and (3) improved coverage compared with the NVSS, if the database also met the first two criteria. Three databases fit these criteria: Fatal Encounters, Mapping Police Violence, and The Counted. A discussion of prominent datasets that we excluded from analysis, including the *Washington Post's* Fatal Force, the National Violent Deaths Reporting System, and the Arrest-Related Deaths Program, is included in the appendix (p 3).

The Fatal Encounters, Mapping Police Violence, and The Counted databases cover all 50 states and Washington, DC (hereafter referred together as “states”), and were compiled by collating news reports and public records requests. The strategy of capturing police violence deaths using publicly available information is known as an open-source methodology.^38^ The open-source databases collectively cover only 20 years from 2000 to 2019, much less than the 39 years of the NVSS that we had available from 1980 to 2018, limiting the scope of accurate estimates of police violence (table). The longest-running open-source database, Fatal Encounters, has a very broad case definition that includes all deaths during encounters with the police, with no requirement of police culpability. Mapping Police Violence and The Counted, which follow a more specific definition of police violence in only considering civilians killed by police, collectively cover only 7 years, from 2013 to 2019.

**Table:** Key facts on included data sources for police violence in the USA

	**Organisation type**	**Years of data available**	**Case definition**	**Data collection method**	**Percentage of cases missing race or ethnicity**	**Sources of bias from gold standard**
Fatal Encounters	501(c)(3) non-profit	2005–19*†	People killed during encounters with the police	Open-source methodology: researchers collate news reports and public records requests	22%	Case definition; percentage missing in race or ethnicity
Mapping Police Violence	501(c)(3) non-profit research collaborative	2013–19*	Police killings	Open-source methodology: researchers collate news reports and public records requests	9%	Percentage missing in race or ethnicity
The Counted	Project of *The Guardian* newspaper	2015–16	People killed by the police and other law enforcement agencies	Open-source methodology: researchers collate news reports and public records requests	2%	Not applicable (gold standard)
National Vital Statistics System	Government system coordinated by the National Center for Health Statistics	1980–2018‡	Legal intervention ICD code as underlying cause of death (Y35.0–Y35.4, Y35.6–Y35.9, and Y89.0 in ICD-10, and E970–E977 in ICD-9§)	Death certificates: medical examiner or coroner determines cause of death	15%	Data collection method; percentage missing in race or ethnicity

ICD=International Classification of Diseases. GBD=Global Burden of Diseases, Injuries, and Risk Factors Study.

* Data collection is ongoing; the last complete year of data at the time of this study was 2019.

† Fatal Encounters includes data for 2000–04, which we chose to exclude due to concerns about their completeness (see appendix pp 6–7).

‡ The National Vital Statistics System existed before 1980; however, we limit our analyses to data from 1980 onwards to enable use of the time series produced by GBD.

§ See appendix pp 23–25 for full code names.

### Data standardisation

We extracted and standardised the age, sex, US state, year of death, and race and ethnicity of each decedent for all data sources. Details on the standardisation of age and sex are included in the appendix (p 4). Race and ethnicity categories reported in the raw data varied across data source, by US state, and over time,^39^ presenting a substantial challenge for standardisation. We chose to standardise the data to the four categories non-Hispanic Black, non-Hispanic White, non-Hispanic of other races, and Hispanic of any race, because they have sufficiently large populations to support our statistical analyses. Within these categories, we considered Indigenous people to be part of non-Hispanic of other races. The separation of race and Hispanic ethnicity is based on the standards for race and ethnicity categorisation maintained by the USA Office of Management and Budget (OMB).^40^ It is important to note that race and ethnicity are social classifications that, while able to affect peoples' lives through social and political forces, have no biological or scientific basis. The OMB has acknowledged the lack of scientific basis for their race and ethnicity categories.^41^

For the open-source databases, we reassigned deaths with unknown race or ethnicity proportionally to the standard categories based on the pattern of deaths with known race and ethnicity in each data source. This approach assumes that within a given dataset, the likelihood of a decedent's race or ethnicity being unknown is independent of their true race and ethnicity. The potential bias introduced by this assumption diminishes as the proportion of deaths with unknown race in a given dataset decreases; therefore, The Counted will have the most accurate race and ethnicity distribution after reassignment, as only 2% of 2239 deaths had unknown race or ethnicity before reassignment (table). The NVSS presented a particular challenge in the handling of deaths with unknown race or ethnicity, since every state is missing ethnicity for more than 90% of deaths for several years starting in 1980, ranging from 3 to 17 years depending on the state (appendix pp 21–22). For these state-years, we calculated the relative rate of police violence between Hispanic and non-Hispanic people for the smallest viable series of succeeding years with more than 50% ethnicity completeness and back-extrapolated this ratio on the basis of ethnicity-specific population estimates. This approach assumes that the relative rate of police violence between Hispanic and non-Hispanic people was constant across the early years of the 1980–2019 time series. Details on the redistribution of unknown race and ethnicity are included in the appendix (pp 4–6).

### Quantifying biases in data sources

From the four included datasets, we considered The Counted to be the gold standard because of its open-source methodology, case definition of police violence, and high completeness on race and ethnicity. We used a network meta-regression (NMR)^42^, ^43^, ^44^, ^45^, ^46^ to quantify the biases of the three other datasets compared with The Counted for any given state, race and ethnicity, and year. The advantage of using NMR is that it uses all available pairwise comparisons between datasets to deduce the relative biases between them: both direct comparisons between the non-standard datasets and The Counted, which can necessarily span only 2 years (2015–16), and indirect comparisons made among the non-standard datasets from the years where they overlap (2005–19). This increases the amount of data available to the meta-regression by 5·5 times.

We specified the NMR as a mixed-effects log-transformed linear regression, with fixed effects β on state (*l*), race and ethnicity (*r*), and percentage of police violence deaths in the NVSS caused by firearms (PF), and a random effect (*u*) on the state, race and ethnicity, and year combination:

(1) $$\begin{array}{c} \ln\left(\frac{{\text{rate}}_{l,y,r}^{x_{1}}}{{\text{rate}}_{l,y,r}^{x_{2}}}\right)=\left(\beta_{0}^{x_{1}}-\beta_{0}^{x_{2}}\right)+\left(\beta_{l}^{x_{2}}-\beta_{l}^{x_{2}}\right)+\left(\beta_{r}^{x_{1}}-\beta_{r}^{x_{2}}\right)\\ +\left(\beta_{\text{PF}}^{x_{1}}-\beta_{\text{PF}}^{x_{2}}\right)\times {\text{PF}}_{l,y,r}+u_{l,y,r}+ɛ \end{array}$$

where rate^x^_l,y,r_ is the cause-specific mortality rate due to police violence for state *l*, year *y*, and race and ethnicity group *r* in dataset *x*, and β^x^_v_ represents the mean effect of variable *v* on the log-mortality ratio of dataset *x* to The Counted. By definition, the β_v_ coefficients for The Counted are zero for all variables *v*. We also impose a prior such that β^x^_PF_=0 for *x*=Fatal Encounters or *x*=Mapping Police Violence. For *x*_1_=NVSS and *x*_2_=The Counted, the quantity estimated by the linear predictor is the log of the reporting rate of the NVSS, assuming that The Counted represents the full burden of fatal police violence. By using this model specification, we assume that systematic biases between all datasets, including under-reporting in the NVSS, vary by state, race, and ethnicity and are constant across age and sex. We assume that the systematic biases between Fatal Encounters, Mapping Police Violence, and The Counted are constant over time from 2005 to 2019, which is well supported by the years in which these sources overlap, but the inclusion of β^NVSS^_PF_ allows under-reporting in the NVSS to vary over time according to this variable. A discussion of our covariate selection process, how we calculated the cause-specific mortality rate, how we offset state-race-ethnicity-years with zero deaths in the raw data to apply the log transform, and why we aggregated age and sex for this analysis is included in the appendix (pp 7–8).

### Producing comparable estimates

To produce comprehensive, comparable police violence estimates, we used the estimates of the under-reporting rates in the NVSS by state and race and ethnicity obtained from the NMR to correct the observed deaths *D* in the NVSS using the following equation:

(2) $$\begin{array}{c} D_{l,y,r}^{\text{NVSS},\text{corr}}=D_{l,y,r}^{\text{NVSS},\text{obs}}\times \exp[-(\beta_{0}^{\text{NVSS}}+\beta_{l}^{\text{NVSS}}+\beta_{r}^{\text{NVSS}}\\ +\beta_{PF}^{\text{NVSS}}\times {\text{PF}}_{l,y,r})] \end{array}$$

We made similar adjustments to Fatal Encounters and Mapping Police Violence using their respective over-reporting and under-reporting rates, due to their differing case definitions and lower race and ethnicity detail compared with The Counted. At this point, we had corrected the data sources so that all four matched the methodology and case definition of our gold-standard source. However, each dataset still represented a distinct observation of the overall problem of police violence and covered only a specific segment of time from 1980 to 2019. To obtain comprehensive estimates of police violence that are comparable across all dimensions from 1980 to 2019 and that include the uncertainty associated with each underlying observation, we ran a predictive model on the NMR-adjusted data sources.

We started with a Poisson regression on time for each state, race, and ethnicity combination:

(3) $$\begin{array}{l} {\text{death}}_{l,y,r}\sim \text{Poisson}\left(\lambda_{l,y,r}\times {\text{population}}_{l,y,r}\right)\\ \text{ln}\left(\lambda_{l,y,r}\right)=\alpha_{l,r}+\beta_{l,r}\times \gamma \end{array}$$

where rate^*x*^_l,y,r_ is the cause-specific mortality rate due to police violence for state *l*, year *y*, and race and ethnicity group *r*. We then used a log-transformed spatiotemporal Gaussian progress regression (ST-GPR), as used in GBD causes of death ensemble modelling (known as CODEm), to incorporate any systematic variation in the data from the initial Poisson model across state, race and ethnicity, and time into the model predictions.^47^ A table of the ST-GPR parameters that we used is included in the appendix (p 28). Finally, we split the estimates from ST-GPR into detailed age bins and by sex using the age-sex pattern of cause-specific mortality in the underlying data and the GBD causes of death age-sex-splitting algorithm.^1^ This splitting was independent of state, year, race, and ethnicity, and therefore assumed a constant age-sex pattern across these dimensions.

### Estimating further race and ethnicity detail at the national level

Within our primary analysis, our statistical methods were unable to accurately analyse smaller race and ethnicity groups than non-Hispanic White, non-Hispanic Black, non-Hispanic of other races, and Hispanic of any race at the state level, primarily due to instability in the offsetting method for observed zeros that we use to apply log transforms. However, these broad categories can hide large disparities in police violence against smaller race and ethnicity groups. In particular, previous research has shown that Indigenous people are killed by the police at higher rates than any group other than Black people.^2^

To address this, we did a secondary analysis in which we produced national-level estimates of police violence deaths for five race and ethnicity groups from 1990 to 2019: Hispanic of any race, non-Hispanic White, non-Hispanic Black, non-Hispanic Indigenous, and non-Hispanic of other races, where the last two categories collectively cover the same races and ethnicities as non-Hispanic of other races in our main analysis. We were only able to do this secondary analysis for 1990–2019 due to a lack of sufficiently detailed population estimates for 1980–89. We began by re-prepping all input data sources with these five race and ethnicity groups (denoted *r*′). We then aggregated the data to the national level to compensate for the smaller populations and ran an NMR to quantify dataset-level biases:

(4) $$\ln\left(\frac{{\text{rate}}_{y,r^{\prime }}^{x_{1}}}{{\text{rate}}_{y,r^{\prime }}^{x_{2}}}\right)=\left(\beta_{0}^{x_{1}}-\beta_{0}^{x_{2}}\right)+\left(\beta_{r^{\prime }}^{x_{1}}-\beta_{r^{\prime }}^{x_{2}}\right)+u_{y,r^{\prime }}+ɛ$$

We did not include percentage of NVSS police violence deaths caused by firearms as a covariate in this regression due to lack of significance at this level of aggregation. We adjusted the data to remove these systematic biases in a method analogous to equation 2 and used the adjusted data to calculate the proportions of decedents in the non-Hispanic of other races group, as defined in our main analysis, that were non-Hispanic Indigenous and non-Hispanic of other races as defined in our secondary analysis. Finally, we used these proportions to split the national-level results from the primary ST-GPR model into final estimates for the five race and ethnicity groups.

### Role of the funding source

This study was funded by the Bill & Melinda Gates Foundation, National Institute of Minority Health and Health Disparities, and the National Heart, Lung, and Blood Institute. Coauthors affiliated with these organisations provided feedback on initial maps and drafts of this manuscript. Otherwise, the funders had no role in study design, data collection, data analysis, data interpretation, writing of the final report, or decision to submit for publication.

## Results

**Figure 1:**
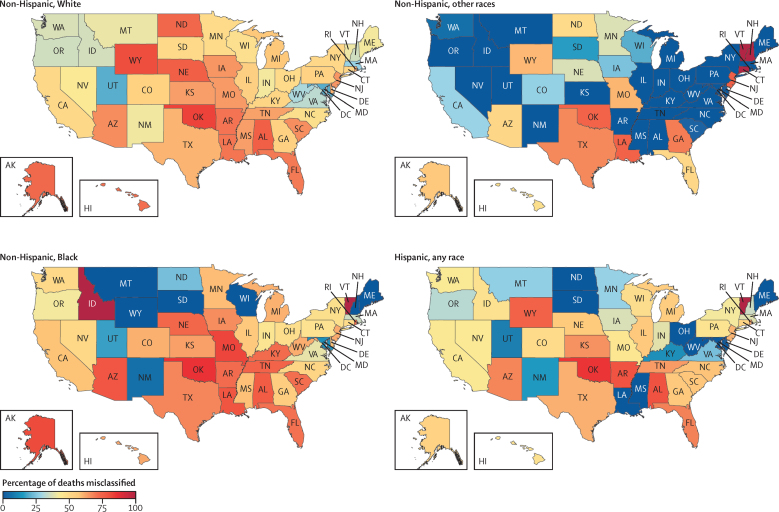
Percentage of police violence deaths misclassified in the NVSS by race, ethnicity, and state in the USA, 1980–2018 Percentages are calculated on the basis of our modelled estimate of the true number of police violence deaths in each race, ethnicity, state, and year. NVSS=National Vital Statistics System.

Compared with our findings, the misclassification of police violence in NVSS data is extensive (figure 1). From 1980 to 2018, the NVSS did not report 17 100 deaths (95% UI 16 600–17 600) out of 30 800 deaths (30 300–31 300) that we estimated, accounting for 55·5% (54·8–56·2) of all police violence deaths from 1980 to 2018. In 2018, the most recent year of NVSS data available to us, there were 642 deaths (596–690) missing out of 1240 total estimated deaths (1190–1290) in our analysis, which is a misclassification of 51·8% (50·0–53·7). The total under-reporting is also disparate by race and ethnicity. From 1980 to 2018, the greatest under-reporting of deaths was among non-Hispanic Black people, with 5670 deaths (5390–5970) missing out of an estimated 9540 total deaths (9260–9830), which is 59·5% (58·3–60·7) misclassified. In this same time period, the NVSS did not record 8540 deaths (8200–8910) out of an estimated 15 200 (14 900–15 600) for non-Hispanic White people, which is a similar 56·1% (55·2–57·2) misclassified; 281 deaths (226–346) out of an estimated 861 (806–925) for non-Hispanic other races, which is 32·6% (28·1–37·4) misclassified; and 2580 deaths (2390–2780) out of an estimated 5170 (4980–5360) for Hispanic people of any race, which is 50·0% (48·1–51·8) misclassified. When we disaggregated non-Hispanic Indigenous people from the remaining non-Hispanic people of other races in our secondary analysis, we found that 63 deaths (44–85) out of an estimated 289 (270–311) total deaths were missing for non-Hispanic Indigenous people from 1990 to 2018, which is 21·6% (16·2–27·2) misclassified, the lowest under-reporting rate of any race and ethnicity group. Under-reporting in the NVSS varies even more widely across US states. From 1980 to 2018, the top five states with the highest under-reporting rates were Oklahoma, with an estimated 83·7% (82·0–85·3) of deaths misclassified; Wyoming, with 79·1% (71·3–85·7); Alabama, with 76·9% (73·9–79·9); Louisiana, with 75·7% (72·8–78·4); and Nebraska, with 72·9% (67·3–77·8). The five states with the lowest under-reporting rates in the same time frame were Maryland, with 16·4% (9·1–23·4) of estimated deaths misclassified; Utah, with 19·8% (9·9–30·1); New Mexico, with 26·4% (18·6–33·4); Massachusetts, with 32·5% (22·5–42·1); and Oregon, with 36·3% (29·7–42·5).

**Figure 2:**
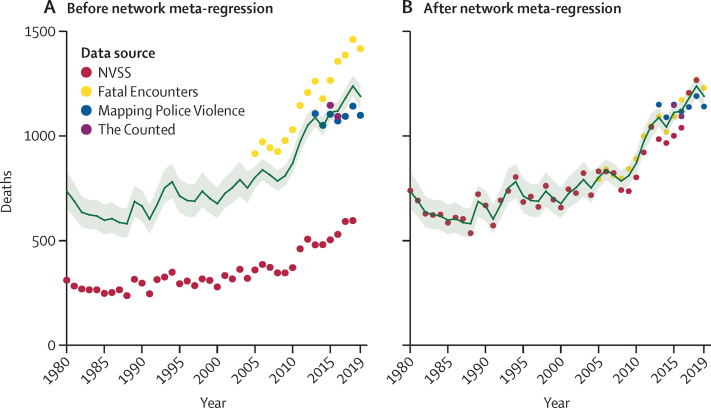
Input data and model estimate for police violence deaths in the USA, 1980–2019 The figure shows the raw data (A) and the data after applying correction factors from the network meta-regression to each data source (B). The corrected data (B) were then used to fit the model estimate shown in green (with shaded 95% uncertainty intervals) using Poisson regression and spatiotemporal Gaussian process regression. NVSS=National Vital Statistics System.

**Figure 3:**
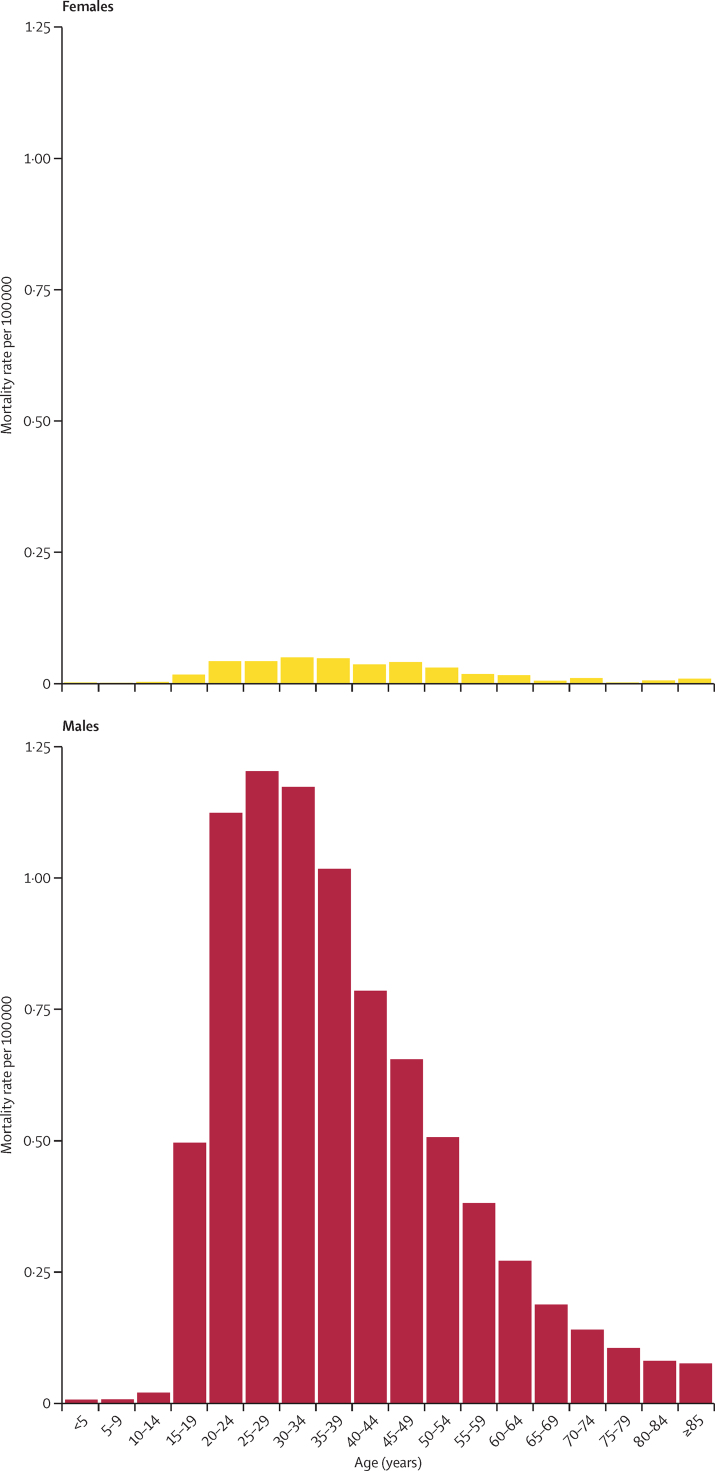
Police violence mortality rate per 100 000 by age and sex in the USA, 1980–2019 Results are from our modelled estimates of police violence, and are shown at the national level for all races and ethnicities due to limitations of our methods.

Once the underlying data were corrected for under-reporting, our predictive model estimated that the total number of deaths from police violence in the USA, for all races and ethnicities and all states from 1980 to 2019, was 32 000 deaths (95% UI 31 500–32 500), with 1190 deaths (1130–1240) in 2019 (figure 2). The total estimated mortality rate for all races and ethnicities in the USA from 1980 to 2019 is thus 0·28 (0·27–0·28) per 100 000 people. When aggregating all races and ethnicities, the age-standardised mortality rate due to police violence was 0·25 (0·24–0·26) per 100 000 in the 1980s and 0·34 (0·34–0·35) per 100 000 in the 2010s, an increase of 38·4% (33·4–45·1). Estimated deaths due to police violence were also orders of magnitude higher for males of any race or ethnicity than females of any race or ethnicity, with 30 600 deaths (30 100–31 000) in males and 1420 deaths (1400–1440) in females from 1980 to 2019, a difference of 2054% (2054–2054; figure 3). In 2019, 1140 police violence deaths (1080–1190) occurred in males and 53 (51–55) occurred in females.

**Figure 4:**
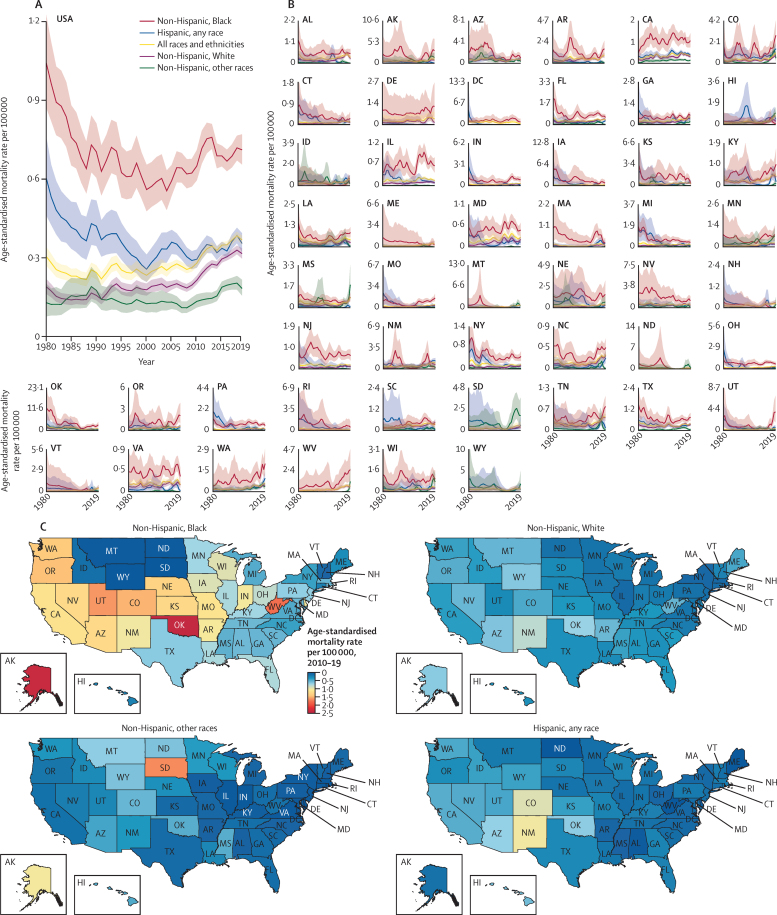
Police violence age-standardised mortality rate per 100 000 in the USA, 1980–2019 All results are from our modelled estimate of police violence. (A) Age-standardised mortality rates by race, ethnicity, and year are shown for the USA and the individual states, with 95% uncertainty intervals shown in the shaded regions. Note that scales are not fixed across states to aid comparisons across race and ethnicity for each state. (C) Age-standardised mortality rate by race, ethnicity, and state, 2010–19.

**Figure 5:**
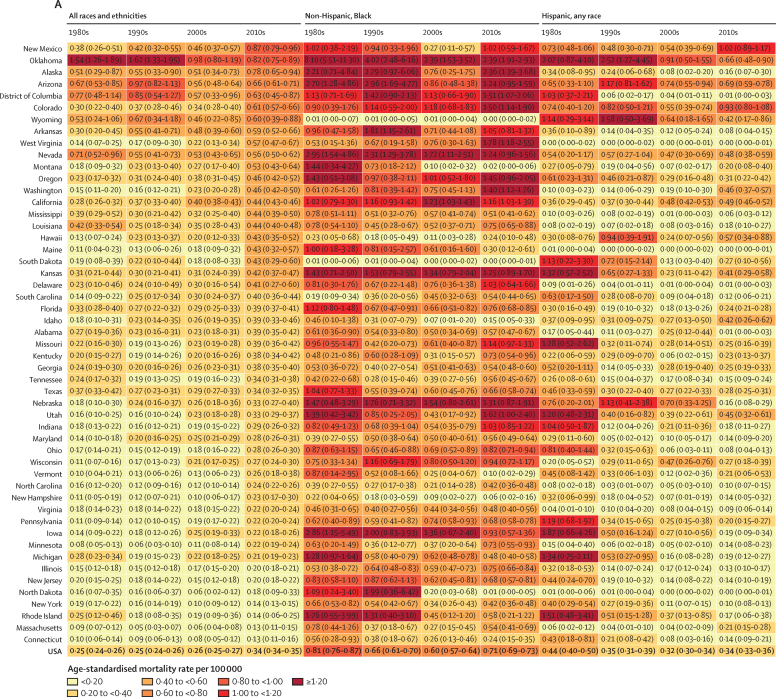

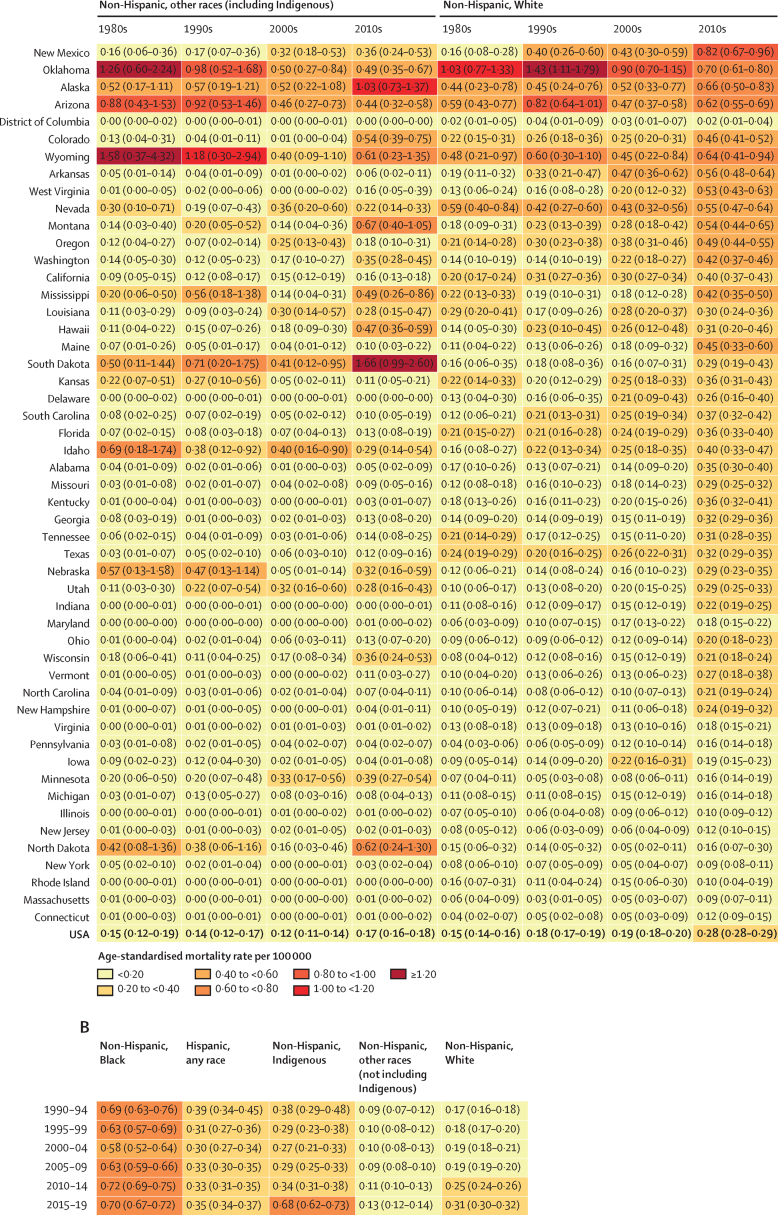
Age-standardised mortality rate due to police violence by race, ethnicity, and decade, 1980–2019 (A), with detailed race and ethnicity breakdown, 1990–2019 (B) (A) Results are from our modelled estimates of police violence. States are presented in descending order of police violence for all races and ethnicities in 2010–19, finishing with the USA total. (B) Results are from our secondary analysis of police violence at the national level by more detailed race and ethnicity groups, estimating non-Hispanic Indigenous people separately from non-Hispanic people of all other races.

The age-standardised mortality rate due to police violence in non-Hispanic Black people from 1980 to 2019 was estimated to be 0·69 (95% UI 0·67–0·71) per 100 000, which was 3·5 times higher than the rate for non-Hispanic White people, at 0·20 (0·20–0·20) per 100 000. Over the same period, the estimated rate in Hispanic people of any race from 1980 to 2019 was 0·35 (0·34–0·36) per 100 000, 1·8 times higher than for non-Hispanic White people, and the rate for non-Hispanic people of other races was 0·15 (0·14–0·16) per 100 000. When restricting data to 1990–2019 to allow a more detailed breakdown of non-Hispanic people of other races, the estimated rate was 0·38 (0·36–0·41) per 100 000 in non-Hispanic Indigenous people, 1·8 times higher than the rate in non-Hispanic White People (0·22 [0·21–0·22]). By contrast, we found fatal police violence rates in non-Hispanic people of other races, when not including non-Hispanic Indigenous people, to be 0·11 (0·10–0·12), considerably lower than for non-Hispanic White people. The estimated age-standardised mortality rate due to police violence for non-Hispanic Black people was higher in every year from 1980 to 2019 compared with non-Hispanic White people (Figure 4, Figure 5). The rate due to police violence of Hispanic people of any race was also higher than their White, non-Hispanic counterparts in every year from 1980 to 2019 (Figure 4, Figure 5).

The temporal trends of fatal police violence have several important features. From 1980 to 1990, non-Hispanic White people, non-Hispanic Black people, and Hispanic people of any race all experienced a reduction in police violence: from 1980 to 1990, the estimated age-standardised mortality rate of police violence decreased by 32·0% (95% UI 15·3 to 46·5) in non-Hispanic Black people, by 16·9% (−1·7 to 31·4) in non-Hispanic White people, and by 29·3% (2·4 to 48·7) in Hispanic people of any race (figure 4). Additionally, there has been an increase in fatal police violence in non-Hispanic White people since 1980 in our estimates (Figure 4, Figure 5). In 1980, the national age-standardised mortality rate due to police violence was 0·19 (95% UI 0·16 to 0·22) for non-Hispanic White people; by 2019, this had increased to 0·32 (0·30 to 0·34). However, overwhelming racial disparities in police violence have remained constant across the entire time series from 1980 to 2019 for non-Hispanic Black people, and have increased for non-Hispanic Indigenous people (Figure 4, Figure 5). In particular, our findings show that the age-standardised mortality rate due to police violence in non-Hispanic Indigenous people spiked from 0·34 (0·31 to 0·38) in 2010–14 to 0·68 (0·62 to 0·73) in 2015–19 (figure 5).

The US states with the highest age-standardised mortality rate of police violence between 1980 and 2019 in our analysis were Oklahoma (1·22 deaths [95% UI 1·10–1·35] per 100 000), the District of Columbia (0·70 [0·55–0·89]), Arizona (0·70 [0·65–0·76]), Alaska (0·60 [0·49–0·73]), Nevada (0·57 [0·51–0·64]), and Wyoming (0·56 [0·40–0·80]; figure 4). Mortality rates by state also illustrate higher rates of police violence towards non-Hispanic Black people in 42 states over the past decade (2010–19) compared with non-Hispanic White people (Figure 4, Figure 5). States with the lowest age-standardised mortality rate of fatal police violence between 1980 and 2019 were Massachusetts (0·08 [0·07–0·09]), Connecticut (0·09 [0·08–0·12]), Minnesota (0·12 [0·10–0·14]), North Dakota (0·14 [0·09–0·22]), New Hampshire (0·14 [0·11–0·18]), and New York (0·15 [0·14–0·16]; Figure 4, Figure 5).

For every decade from 1980 to 2019, the highest age-standardised mortality rate due to police violence by state occurred in non-Hispanic Black people. In 2010–19, the states with the highest rates of police violence towards non-Hispanic Black Americans were Oklahoma, Alaska, West Virginia, Utah, and the District of Columbia, whereas in 2000–09, the states with the highest rates were Oklahoma, Nevada, Nebraska, Iowa, and Kansas. Oklahoma has among the highest rates of police violence against non-Hispanic Black Americans in the country, with a peak estimated age-standardised mortality rate of 12·20 deaths (5·36–23·19) per 100 000 in 1980 and a current rate of 3·05 deaths (1·82–4·59) per 100 000 in 2019. This aligns with extremely high rates of police violence in recent years as reported in the open-source databases, high under-reporting rates in the NVSS, and large peaks in police violence in the 1980s and 1990s in the NVSS.

## Discussion

Our analysis of police violence in the USA shows that the NVSS misclassified and subsequently under-reported 55·5% (95% UI 54·8–56·2) of our estimated deaths from police violence between 1980 and 2018. Consistent with the estimated rates of fatal police violence in this group, the highest under-reporting in the NVSS occurred for deaths of Black Americans at 59·5% (58·3–60·7). However, the issue of under-reporting does not only affect Black Americans. The NVSS missed 56·1% (55·2–57·2) of deaths of non-Hispanic White people, 32·6% (28·1–37·4) of non-Hispanic people of other races, and 50·0% (48·1–51·8) of Hispanic people of any race. The police have disproportionately killed Black people at a rate of 3·5 times higher than White people, and have killed Hispanic and Indigenous people disproportionately as well. The rate of fatal police violence was higher in every year for Black Americans than for White Americans. In the USA, more males died from police violence in 2019 (1140 deaths [95% UI 1080–1190]) than from environmental heat and cold exposure (931 deaths [891–971]), Hodgkin lymphoma (835 deaths [776–1013]), testicular cancer (486 deaths [455–537]), appendicitis (373 deaths [234–545]), exposure to forces of nature (62 deaths [56–68]) and sexually transmitted diseases (37 deaths [95% UI 33–44]).^1^

In the USA, NVSS data under-report police violence compared with all open-source data, including those using a similar case definition, such as Mapping Police Violence**.** Previous research has found that open-source methods can independently capture the majority of police violence deaths and accurately capture demographic information on decedents.^38^, ^48^ The USA Bureau of Justice Statistics considers such methods preferable to government reporting systems due to issues of official under-reporting.^38^ A study examining under-reporting of the NVSS linked deaths from The Counted to identifiable NVSS records for 2015 and estimated that 1166 deaths occurred in the USA in 2015. The NVSS captured only 524 of these deaths, while The Counted captured 1086.^20^ Our study found that the NVSS undercounted 607 deaths (95% UI 569–646) in 2015, capturing only 504 compared with our estimate of 1110 (1070–1150).

Physicians are typically responsible for filling out the cause of death section of the death certificate, but state laws require that a medical examiner or coroner do so for homicides or cases where there is suspicion of crime or foul play, including police violence.^19^, ^35^ However, only some cities have forensic pathologists to act as the coroner, and in small, rural counties, the coroner can be a physician with no forensic training, the sheriff, or a mortician. The text fields of the cause of death section are filled out by the certifier; these responses are then translated into International Classification of Diseases (ICD) codes by software and nosologists using WHO's published code selection rules.^50^ According to these rules, deaths due to police violence should be classified under the legal intervention codes, which are defined as “injuries inflicted by the police or other law-enforcing agents, including military on duty, in the course of arresting or attempting to arrest lawbreakers, suppressing disturbances, maintaining order, and other legal action”.^51^ In cases of police violence, many text fields contribute to the coding process, including the causal chain indicating the full sequence of events leading to death and the manner of death section. One text field is particularly crucial: a section that, in case of injury, asks the certifier to “describe how the injury occurred”. If this section does not mention that the decedent was killed by the police, then the death will not be assigned to legal intervention.^18^, ^19^

Previous studies have documented that the death certification system regularly under-reports deaths due to legal intervention.^18^, ^19^, ^20^, ^21^, ^48^ The under-reporting is related to several factors, including the coroner or medical examiner failing to indicate police involvement in the text fields of the death certificate's cause of death section or errors in the process of assigning ICD codes even when the death certificate shows police involvement.^20^ There is considerable evidence that omission of police involvement from the description of how the injury occurred is responsible for the misclassification of police violence as homicides.^19^, ^20^ A police violence death might be misclassified as another cause because the certifier fails to mention the police in the “describe how the injury occurred” section, or because the certificate is incorrectly coded after the fact.^20^ The “describe how the injury occurred” section is open-ended and comes with no explicit instructions to mention police involvement,^20^ and a certifier might lack the knowledge or training to fill out the form correctly. There are also substantial conflicts of interest within the death investigation system that could disincentivise certifiers from indicating police involvement, including the fact that many medical examiners and coroners work for or are embedded within police departments.^52^ In a web-based survey of National Association of Medical Examiners members in 2011, 22% of respondents reported having been pressured by an elected official or appointee to change cause or manner of death on a certificate.^53^ If this systematic under-reporting is occurring globally—which seems probable^54^—then using open-source databases that report police violence without the biases reflected in government reporting agencies is a crucial public health need in the USA and potentially globally as well.

Under-reporting obfuscates and minimises the larger public health issue, high rates of fatal police violence with serious disparities in race and ethnicity. While the purpose of our model was strictly predictive, and not designed or intended for inferential or causal analysis, it is crucial to consider the causes of police violence to understand this health crisis. Long-standing research in the USA has well established that the disproportionate amount of police violence against Black Americans is driven by systemic racism.^2^, ^55^, ^56^ Black Americans experience disproportionately high levels of police contact, even for crimes that Black and White Americans commit at the same rates, such as certain drug offences, and for interactions that are not triggered by criminal activity, such as investigatory traffic stops.^57^ Police are more likely to shoot Black civilians than White civilians given the same levels of criminal activity, even when the civilian is unarmed.^58^, ^59^, ^60^ In addition to a disproportionate burden of fatal violence at the hands of the police, systemic racism also makes non-Hispanic Black people more likely to be incarcerated than other racial groups (details on the association between incarceration and police violence can be found in the appendix p 9).^61^, ^62^

Racial bias in policing does not exist in a vacuum: it follows the pattern of anti-Black racism in the criminal justice system throughout the USA's history.^57^ Police forces should exist to enforce laws that protect public safety, but throughout the USA's history, police have been used to enforce racist and exploitative social orders that endanger the safety of the most marginalised groups in society.^63^ Some of the earliest examples of policing include the capture of runaway slaves, dismantling labour strikes and movements, and stopping riots, protests, or other expressions of social rage.^63^, ^64^, ^65^, ^66^, ^67^ In the post-slavery South, the police stopped organisers, threatened and beat protestors, denied protest permits, and did not protect demonstrators from mobs that bombed and killed them.^63^, ^68^, ^69^ Today, US police are heavily militarised, and fatal police violence disproportionately affects Black, Indigenous, and Hispanic people. Police are trained that any interaction can turn deadly and that they should react as such.^63^ Heavily armed officers can dangerously escalate situations that never needed violent intervention.^70^, ^71^, ^72^ Federal programmes provide the police with military equipment and outfit officers with lethal weapons that are unnecessary to protect their communities.^70^ Importantly, deaths from police violence are seldom uniformly distributed within populations, frequently serving to exacerbate societal unrest and highlighting persistent inequalities.^72^ During the George Floyd protests in 2020, which were in direct response to racist police violence, *The Guardian* documented 950 instances of police violence against civilians and journalists.^73^ These include more than 500 instances of the police using less-lethal rounds (rubber bullets), pepper spray, and tear gas; 60 instances of unlawful assembly to arrest protesters; and 19 of permissiveness to white supremacists,^73^ when not showing the same restraint towards demonstrators. Accountability and transparency in policing are lacking, as evidenced by ongoing problems with under-reporting. Police officers who kill civilians are rarely charged with a crime; Mapping Police Violence reports that in 2017, of 1147 deaths, officers were charged with a crime in 13 cases, or 1% of the time. Police violence and racism in policing in the USA are not new or unexplained problems; they are the current manifestations of a system that was built to uphold racial hierarchy for most of the USA's history.

This study has some limitations. First, this study does not calculate or address non-fatal injuries inflicted by the police. This topic is crucial to understanding the full burden of police violence and should be examined in future studies.^74^, ^75^ We also excluded police officers killed by civilians and executions from our analysis of police violence. These data can be found elsewhere^76^ and should be analysed separately. This analysis does not include military police and residents who might have been harmed by military police, in the USA or abroad. Additionally, the open-sourced data used for this study do not cover fatal police violence in US territories; therefore, violence in those locations would need to be analysed separately. Finally, our modelling framework assumes constant under-reporting in NVSS across age and sex, an assumption that might not hold in all cases (appendix p 8), and our estimation strategy applies national-level age and sex trends to all states and races. We encourage future research to improve the estimation of police violence by age and sex.

Our data processing of demographic information also had several limitations. Death certificates in the USA only allow for a binary designation of sex and do not distinguish between sex assigned at birth and gender identity; by contrast, the open-source databases all record several decedents with their gender listed as transgender or non-binary. To tabulate our data to populations large enough to support our statistical analysis, we used the GBD's age-sex-splitting algorithm to reassign deaths with transgender or non-binary coding in the three open-source databases to male and female. This approach erases the existence of non-cisgender people and masks the disproportionately high rates of violence against transgender and, most acutely, Black transgender people.^77^, ^78^ The intersectionality of gender, race and ethnicity, sexual orientation, and other identities and the relationship to fatal police violence should be studied in the future.^79^

Due to high missingness in race and ethnicity in early years of Fatal Encounters and NVSS data, we relied on algorithms such as proportional reassignment based on known data, surname-based and geography-based imputation, and back-extrapolation of relative mortality rates to obtain estimates by race and ethnicity. Although these methods rely on imperfect assumptions, we believe that they are as correct as possible without record linkage or further primary data collection and that they allow us to accurately estimate serious disparities in police violence across race and ethnicity.

Police violence, like other forms of violence, is preventable.^13^, ^80^ The American Public Health Association, the American College of Physicians, the American Medical Association, the Institute for Health Metrics and Evaluation, as well as a large number of government agencies, advocacy groups, and community organisations, condemn police violence and its underlying racism, identifying it as a public health crisis.^6^, ^12^, ^14^, ^81^, ^82^

Data collection that occurs without conflicting interests of the state is crucial to capture the full burden of deaths due to police violence. With each iteration of GBD, the Institute for Health Metrics and Evaluation will compare open-source data with NVSS data and publicise the findings—a step towards transparent reporting of fatal police violence. Some progress has been made in the USA with the implementation of the National Violent Deaths Reporting System in 2003, a state-based surveillance system that shows improved coverage of fatal police violence when compared with the NVSS; however, this system still shows undercounting when compared with open-source databases and needs improved geographical and temporal coverage (appendix p 3). Globally, some independent data-collection agencies are already reporting on state-sanctioned violence, including Amnesty International, The Armed Conflict Location & Event Data Project, and the Uppsala Conflict Data Program. These data sources already critically inform GBD estimates for police conflict and executions, war and terrorism, and other violent causes. We suggest that other researchers adapt our data-seeking criteria and correction methodology to study biases that might exist in other government-run data-collection agencies globally. The increased use of open-source data-collection initiatives allows researchers and policy makers to document and highlight disparities in police violence by race, ethnicity, and gender, allowing for targeted, meaningful changes to policing and public safety that will prevent loss of life.

Improved training and clearer instructions on how to document police violence in text fields on death certificates could improve reporting.^35^ Coroners and forensic medical experts also propose that to avoid incorrect assignment of cause of death due to pressure from the police, politicians, or the deceased family members, forensic pathologists should work independently from law enforcement.^53^ Additionally, forensic pathologists often must investigate and testify in cases of police violence.^53^ To ensure that pathologists are free from pressures that could influence these cases, pathologists should be awarded whistleblower protections under the law.^53^

Beyond the issue of reporting police violence accurately, the field of public health must turn its attention to ultimately eliminating the burden of police violence. Evidence suggests that there have been some successful reforms to reduce police violence from 1970 to 1985; 50 cities with populations larger than 250 000 residents halved their fatal police violence from 353 to 172 per year, primarily through banning shooting of non-violent fleeing suspects.^83^, ^84^ These decreasing trends are reflected in our temporal results. However, more recent reform efforts to prevent police violence in the USA, including body cameras, implicit bias training, de-escalation, and diversifying police forces, have all failed to further meaningfully reduce police violence rates.^63^, ^85^, ^86^ As our analysis shows, fatal police violence rates and the large racial disparities in fatal police violence have remained largely unchanged or have increased since 1990.

Evidence-based research and advocacy are needed to find solutions that work. The George Floyd protests and the Black Lives Matter movements have opened discussions among the public, media, and health and justice authorities for new strategies, including divestment from police and corresponding investment in evidence-based community resources for violence prevention.^87^ In 2020, Minneapolis announced a US$8 million cut from its police budget,^88^ New York City announced a roughly $1 billion cut from its police budget,^89^ and Seattle announced a $100 million investment in Black and Indigenous communities.^90^ Many other communities are showing investments in evidence-based interventions for communities outside the police. Eugene, Oregon, deploys mental health crisis workers and emergency medical technicians to avoid the use of law enforcement whenever possible.^91^ The Massachusetts House passed a bill in August, 2020, that will revoke qualified immunity for police officers who are “decertified from poor conduct”.^92^ Although it might seem drastic to many in the USA to defund, disarm, or abolish militarised police, there are many places where living without militarised police is already a reality. 19 nations, including Norway and the UK, do not arm their police officers^93^ or only arm select officers. The difference these practices have on loss of life is staggering: no one died from police violence in Norway in 2019, and three people were recorded to have died in England and Wales from police violence between 2018 and 2019.^94^ To respond to this public health crisis, the USA must replace militarised policing with evidenced-based support for communities, prioritise the safety of the public, and value Black lives.

Correspondence to: Prof Mohsen Naghavi, Department of Health Metrics Sciences, Institute for Health Metrics and Evaluation, School of Medicine, University of Washington, Seattle, WA 98105, USA **nagham@uw.edu**

## Data sharing

This study follows the GATHER. To download the data used in these analyses, please visit the Global Health Data Exchange website for data on the NVSS, and Fatal Encounters, Mapping Police Violence, The Counted, and the National Prisoner Statistics Program for all other data.

## Declaration of interests

M Mahmoudi is the co-founder and director of the Academic Parity Movement, a non-profit organisation dedicated to addressing academic discrimination, violence and incivility. K M Mehta reports leadership or fiduciary role in board, society, committee, or advocacy group, paid or unpaid, with the Youth Community Service as a board member, outside the submitted work. J A Singh reports consulting fees from Crealta/Horizon, Medisys, Fidia, Two Labs, Adept Field Solutions, Clinical Care options, Clearview Healthcare Partners, Putnam Associates, Focus Forward, Navigant Consulting, Spherix, MedIQ, UBM LLC, Trio Health, Medscape, WebMD, and Practice Point Communications, and the National Institutes of Health and the American College of Rheumatology; payment or honoraria for lectures, presentations, speakers bureaus, manuscript writing, or educational events from Simply Speaking; support for attending meetings or travel from OMERACT, an international organisation that develops measures for clinical trials and receives arm's length funding from 12 pharmaceutical companies, when traveling bi-annually to OMERACT meetings; leadership or fiduciary role in other board, society, committee or advocacy group, paid or unpaid, with OMERACT as a member of the steering committee, with the US Food and Drug Administration Arthritis Advisory Committee, with the Veterans Affairs Rheumatology Field Advisory Committee as a member, and with the UAB Cochrane Musculoskeletal Group Satellite Center on Network Meta-analysis as a director and editor; stock or stock options in TPT Global Tech, Vaxart Pharmaceuticals, and Charlotte's Web Holdings; and previously owned stock options in Amarin, Viking, and Moderna, all outside the submitted work.

## References

[1] Vos T, Lim SS, Abbafati C (2020). Global burden of 369 diseases and injuries in 204 countries and territories, 1990–2019: a systematic analysis for the Global Burden of Disease Study 2019. Lancet.

[2] Edwards F, Lee H, Esposito M (2019). Risk of being killed by police use of force in the United States by age, race-ethnicity, and sex. Proc Natl Acad Sci USA.

[3] DeGue S, Fowler KA, Calkins C (2016). Deaths due to use of lethal force by law enforcement: findings from the national violent death reporting system, 17 U.S. states, 2009–2012. Am J Prev Med.

[4] Paull J (2019). The use of lethal force by police in the USA: mortality metrics of race and disintegration (2015–2019). J Soc Dev Sci.

[5] Fagan JA, Campbell AD (2020). Race and reasonableness in police killings. Boston Univ Law Rev.

[6] Serchen J, Doherty R, Atiq O, Hilden D (2020). Racism and health in the United States: a policy statement from the American College of Physicians. Ann Intern Med.

[7] Robert Wood Johnson Foundation (October 2017). Discrimination in America: experiences and views of African Americans. https://media.npr.org/assets/img/2017/10/23/discriminationpoll-african-americans.pdf.

[8] Robert Wood Johnson Foundation (November, 2017). Discrimination in America: experiences and views of Native Americans. https://legacy.npr.org/documents/2017/nov/NPR-discrimination-native-americans-final.pdf.

[9] Robert Wood Johnson Foundation (October, 2017). Discrimination in America: experiences and views of Latinos. https://legacy.npr.org/documents/2017/oct/discrimination-latinos-final.pdf.

[10] Herstory Black Lives Matter. https://blacklivesmatter.com/herstory.

[11] Benjamin GC APHA calls out police violence as a public health crisis. https://www.apha.org/news-and-media/news-releases/apha-news-releases/2020/apha-calls-out-police-violence.

[12] Institute for Health Metrics and Evaluation (June 4, 2020). Racism is a public health issue. http://www.healthdata.org/about/racism-public-health-issue.

[13] Haskins J (2018). Why ending police violence is a public health issue: a Q&A with APHA's Georges Benjamin. Nations Health.

[14] American Public Health Association (Nov 13, 2018). Violence is a public health issue: public health is essential to understanding and treating violence in the US American Public Health Association. https://apha.org/policies-and-advocacy/public-health-policy-statements/policy-database/2019/01/28/violence-is-a-public-health-issue.

[15] Global Burden of Disease 2016 Injury Collaborators (2018). Global mortality from firearms, 1990–2016. JAMA.

[16] Potvin L (2020). Black lives matter in Canada too!. Can J Public Health.

[17] Beardsley E (June 4, 2020). ‘No justice in France, either’: French protest police killings in US and at home. NPR. https://www.npr.org/2020/06/04/869877701/no-justice-in-france-either-french-protest-police-killings-in-u-s-and-at-home.

[18] Loftin C, Wiersema B, McDowall D, Dobrin A (2003). Underreporting of justifiable homicides committed by police officers in the United States, 1976-1998. Am J Public Health.

[19] Loftin C, McDowall D, Xie M (2017). Underreporting of homicides by police in the United States, 1976–2013. Homicide Stud.

[20] Feldman JM, Gruskin S, Coull BA, Krieger N (2017). Quantifying underreporting of law-enforcement-related deaths in United States vital statistics and news-media-based data sources: a capture-recapture analysis. PLoS Med.

[21] Hemenway D, Azrael D, Conner A, Miller M (2019). Variation in rates of fatal police shootings across US States: the role of firearm availability. J Urban Health.

[22] Instituto Nacional de Estadística, Geografía e Informática Mortalidad. https://www.inegi.org.mx/programas/mortalidad/default.html.

[23] Instituto Brasileiro de Geografia e Estatística Pesquisa Estatísticas do Registro Civil. Tabela 2685: óbitos, ocorridos no ano, por estado civil, natureza do óbito, sexo, idade, local de ocorrência e lugar do registro. https://sidra.ibge.gov.br/tabela/2685.

[24] PERU Instituto Nacional de Estadística e Informática INEI Población y vivienda. https://www.inei.gob.pe/estadisticas/indice-tematico/poblacion-y-vivienda/.

[25] Australian Bureau of Statistics (May 3, 2011). https://www.abs.gov.au/AUSSTATS/abs@.nsf/Lookup/3303.0Main+Features12009?OpenDocument.

[26] Folkehelseinstitutte The cause of death register's statistics bank. http://statistikkbank.fhi.no/dar/.

[27] Socialstyrelsen Cause of death register. The National Board of Health and Welfare. https://www.socialstyrelsen.se/statistik-och-data/register/alla-register/dodsorsaksregistret/.

[28] Istat Statistics. https://dati.istat.it/Index.aspx.

[29] International Institute for Vital Registration and Statistics (1979).

[30] MEASURE Evaluation Completeness of vital registration (births and deaths). https://www.measureevaluation.org/his-strengthening-resource-center/country-profiles-1/indicators-of-the-status-of-a-health-information-system.html.

[31] Phillips DE, Lozano R, Naghavi M (2014). A composite metric for assessing data on mortality and causes of death: the vital statistics performance index. Popul Health Metr.

[32] Mikkelsen L, Phillips DE, AbouZahr C (2015). A global assessment of civil registration and vital statistics systems: monitoring data quality and progress. Lancet.

[33] Centers for Disease Control and Prevention National Vital Statistics System. https://www.cdc.gov/nchs/nvss/index.htm.

[34] National Research Council (2009).

[35] Adeyinka A, Bailey K (2020).

[36] Institute for Health Metrics and Evaluation (2020).

[37] Stevens GA, Alkema L, Black RE (2016). Guidelines for Accurate and Transparent Health Estimates Reporting: the GATHER statement. PLoS Med.

[38] Bureau of Justice Statistics (July, 2019). Arrest-related deaths program: pilot study of redesigned survey methodology. https://www.bjs.gov/index.cfm?ty=pbdetail&iid=6626.

[39] National Center for Health Statistics (2003).

[40] National Institutes of Health Office of Management and Budget (OMB) Standards. Office of Research on Women's Health. https://orwh.od.nih.gov/toolkit/other-relevant-federal-policies/OMB-standards.

[41] Kaplan JB, Bennett T (2003). Use of race and ethnicity in biomedical publication. JAMA.

[42] Zheng P, Barber R, Sorensen R, Murray C, Aravkin A (2021). Trimmed constrained mixed effects models: formulations and algorithms. J Comput Graph Stat.

[43] IHME Math Sciences (2020). CrossWalk. https://github.com/ihmeuw-msca/CrossWalk.

[44] Murray CJL, Aravkin AY, Zheng P (2020). Global burden of 87 risk factors in 204 countries and territories, 1990–2019: a systematic analysis for the Global Burden of Disease Study 2019. Lancet.

[45] Liu Y, Wang W, Zhang AB, Bai X, Zhang S (2016). Epley and Semont maneuvers for posterior canal benign paroxysmal positional vertigo: a network meta-analysis. Laryngoscope.

[46] White IR, Barrett JK, Jackson D, Higgins JP (2012). Consistency and inconsistency in network meta-analysis: model estimation using multivariate meta-regression. Res Synth Methods.

[47] Foreman KJ, Lozano R, Lopez AD, Murray CJ (2012). Modeling causes of death: an integrated approach using CODEm. Popul Health Metr.

[48] Feldman JM, Gruskin S, Coull BA, Krieger N (2017). Killed by police: validity of media-based data and misclassification of death certificates in Massachusetts, 2004–2016. Am J Public Health.

[50] Centers for Disease Control and Prevention (Feb 13, 2020). International Classification of Diseases, tenth revision (ICD-10). https://www.cdc.gov/nchs/icd/icd10.htm.

[51] International Classification of Diseases tenth revision, Version: 2019. Y35: legal intervention. https://icd.who.int/browse10/2019/en#/Y35.

[52] Singh M (July 3, 2020). http://www.theguardian.com/us-news/2020/jul/02/autopsies-police-killings-medical-misleading.

[53] Melinek J, Thomas LC, Oliver WR, Schmunk GA, Weedn VW (2013). The National Association of Medical Examiners Ad Hoc Committee on Medical Examiner Independence. National Association of Medical Examiners Position Paper: medical examiner, coroner, and forensic pathologist independence. Acad Forensic Pathol.

[54] Amnesty International Police brutality. https://www.amnesty.org/en/what-we-do/police-brutality/.

[55] Peeples L (2019). What the data say about police shootings. Nature.

[56] Schwartz SA (2020). Police brutality and racism in America. Explore (NY).

[57] The Sentencing Project (2018).

[58] Plant EA, Peruche BM (2005). The consequences of race for police officers' responses to criminal suspects. Psychol Sci.

[59] Moore-Berg S, Karpinski A, Plant EA (2017). Quick to the draw: how suspect race and socioeconomic status influences shooting decisions. J Appl Soc Psychol.

[60] Scott K, Ma DS, Sadler MS, Correll J (2017). A social scientific approach toward understanding racial disparities in police shooting: data from the Department of Justice (1980–2000). J Soc Issues.

[61] Zeng Z (March, 2020). Jail inmates in 2018. NCJ 2530442020. https://www.bjs.gov/content/pub/pdf/ji18.pdf.

[62] Nellis A (June 14, 2016). The color of justice: racial and ethnic disparity in state prisons. The Sentencing Project. https://www.sentencingproject.org/publications/color-of-justice-racial-and-ethnic-disparity-in-state-prisons/.

[63] Vitale AS (2017).

[64] Williams H, Murphy PV (1990).

[65] NPR (June 4, 2020). Throughline: American police. https://www.npr.org/2020/06/03/869046127/american-police.

[66] Modak R (Aug 9, 2020). Police unions are anti-labor. Harvard Political Review. https://harvardpolitics.com/police-unions-are-anti-labor/.

[67] Potter G (2013).

[68] Constitutional Rights Foundation Social protests. https://www.crf-usa.org/black-history-month/social-protests.

[69] Equal Justice Initiative (2020). Reconstruction in America.

[70] American Civil Liberties Union Police militarization. https://www.aclu.org/issues/criminal-law-reform/reforming-police/police-militarization.

[71] Cooper HL (2015). War on drugs policing and police brutality. Subst Use Misuse.

[72] Mummolo J (2018). Militarization fails to enhance police safety or reduce crime but may harm police reputation. Proc Natl Acad Sci USA.

[73] Gabbatt A, Thomas T, Barr C (Oct 29, 2020). https://www.theguardian.com/us-news/2020/oct/29/us-police-brutality-protest.

[74] de Brito D, Challoner KR, Sehgal A, Mallon W (2001). The injury pattern of a new law enforcement weapon: the police bean bag. Ann Emerg Med.

[75] Wahl P, Schreyer N, Yersin B (2006). Injury pattern of the Flash-Ball, a less-lethal weapon used for law enforcement: report of two cases and review of the literature. J Emerg Med.

[76] Federal Bureau of Investigation Law Enforcement Officers Killed and Assaulted (LEOKA) Program. https://www.fbi.gov/services/cjis/ucr/leoka.

[77] James SE, Herman JL, Rankin S, Keisling M, Mottet L, Anafi M (2016).

[78] Human Rights Campaign Fatal violence against the transgender and gender non-conforming community in 2020. https://www.hrc.org/resources/violence-against-the-trans-and-gender-non-conforming-community-in-2020.

[79] Heard E, Fitzgerald L, Wigginton B, Mutch A (2020). Applying intersectionality theory in health promotion research and practice. Health Promot Int.

[80] Krug EG, Dahlberg LL, Mercy JA, Zwi AB, Lozano R (2002).

[81] Ehrenfeld JM, Harris PA (May 29, 2020). https://www.ama-assn.org/about/leadership/police-brutality-must-stop.

[82] American Public Health Association Racism is a public health crisis. https://www.apha.org/topics-and-issues/health-equity/racism-and-health/racism-declarations.

[83] Sherman LW (2018). Reducing fatal police shootings as system crashes: research, theory, and practice. Annu Rev Criminol.

[84] Dovey R (July 9, 2018). To stop fatal police shootings, cities should look to the 1980s. Next City. https://nextcity.org/daily/entry/to-stop-fatal-police-shootings-cities-should-look-to-the-80s.

[85] Zuckermann E (June 3, 2020). Why filming police violence has done nothing to stop it. MIT Technology Review. https://www.technologyreview.com/2020/06/03/1002587/sousveillance-george-floyd-police-body-cams/.

[86] Underhill SM (July 6, 2020). Decades of failed reforms allow continued police brutality and racism. The Conversation. http://theconversation.com/decades-of-failed-reforms-allow-continued-police-brutality-and-racism-141011.

[87] Rummler O (Oct 1, 2020). The major police reforms enacted since George Floyd's death. Axios. https://www.axios.com/police-reform-george-floyd-protest-2150b2dd-a6dc-4a0c-a1fb-62c2e999a03a.html.

[88] Schneider A (Dec 10, 2020). Minneapolis shifts $8 million in police funding, but keeps force at current level. NPR. https://www.npr.org/2020/12/10/944938471/minneapolis-shifts-8-million-in-police-funding-but-keeps-force-at-current-level.

[89] Rubinstein D, Mays JC (June 30, 2020). https://www.nytimes.com/2020/06/30/nyregion/nypd-budget.html.

[90] Derrick A (Nov 23, 2020). Mayor Durkan issues statement on council's adoption of 2021 budget. Office of the Mayor. https://durkan.seattle.gov/2020/11/mayor-durkan-issues-statement-on-councils-adoption-of-2021-budget/.

[91] White Bird Clinic (Oct 29, 2020). What is CAHOOTS?. https://whitebirdclinic.org/what-is-cahoots/.

[92] (July 20, 2020). The 192nd General Court of the Commonwealth of Massachusetts. Amendment H.4860. https://malegislature.gov/Bills/191/H4860.

[93] Godin M (June 19, 2020). What the US can learn from countries where cops don't carry guns. Time. https://time.com/5854986/police-reform-defund-unarmed-guns/.

[94] Statista (September, 2019). Number of fatal shootings by Police in England and Wales from 2004/05 to 2018/19. https://www.statista.com/statistics/319246/police-fatal-shootings-england-wales/.

