# Supplementary material for: Fatal police violence by race and state in the USA, 1980–2019: a network meta-regression
Source: Lancet. 2021 Oct 2;398(10307):1239–55. doi: 10.1016/S0140-6736(21)01609-3 (PMC8485022; doi:10.1016/S0140-6736(21)01609-3)
Supplement: Supplementary appendix [file mmc1.pdf]

THE LANCET

Supplementary appendix

This appendix formed part of the original submission and has been peer reviewed. We post it as supplied by the authors.

Supplement to: GBD 2019 Police Violence US Subnational Collaborators. Fatal police violence by race and state in the USA, 1980–2019: a network meta-regression. *Lancet* 2021; **398:** 1239–55.

Supplemental Appendix: Fatal police violence by race and state in the United States, 1980–2019: a network meta-regression for correcting underreporting

Contents

Excluded datasets 3

Biases in open-source data sources 3

Background on NVSS 3

Contestation of non-firearm deaths 4

Details on data standardisation 4

- Age 4
- Sex 4
- Race and ethnicity 4
  - Handling unknown race & ethnicity 4
  - Unknown ethnicity in NVSS 5
  - Unknown race/ethnicity in Fatal Encounters 5
  - Standardizing race/ethnicity groups 6
- Special handling of Fatal Encounters 6
  - Causes of death in Fatal Encounters 6
  - Completeness of Fatal Encounters 6

Modelling 7

- Estimating population 7
- Dealing with zeros 7
- Covariate selection 7
- Modelling assumptions 8
- Estimating age and sex 8
- Raking in ST-GPR 8
- Calculating uncertainty 8
- Sensitivity analyses 9

Incarceration 9

References 9

Compliance with the Guidelines for Accurate and Transparent Health Estimates Reporting (GATHER) 13

Supplemental figures 14

- Appendix Figure 1. Distribution of specified and unspecified race/ethnicity in Fatal Encounters, before and after including imputed race/ethnicity 14
- Appendix Figure 2. Race/ethnicity composition of Fatal Encounters and NVSS, before and after including imputed race/ethnicity in Fatal Encounters 15

Appendix Figure 3. Normalised deaths in Fatal Encounters and NVSS 16
Appendix Figure 4. Ratios of mortality rates for all non-zero data points, pre- and post-offset 17
Appendix Figure 5. Distributions of ratios of mortality rates, pre- and post-offset 18
Appendix Figure 6. Police violence and incarceration rates per 100,000 by state, race, ethnicity, and year, 1980-2018 19
Appendix Figure 7: Methods for the estimation of police violence in the USA by state, race, and ethnicity, 1980-2019 20
Supplemental Tables 21
Appendix Table 1. Percent of deaths missing ethnicity in NVSS by state and year, 1980-1999 21
Appendix Table 2. Legal intervention ICD 10 codes used to identify police violence in NVSS 23
Appendix Table 3. Legal intervention ICD 9 codes used to identify police violence in NVSS 25
Appendix Table 4. Race/ethnicity group standardisation across data sources 26
Appendix Table 5. Causes of death in Fatal Encounters, 2005–2019 28
Appendix Table 6. Spatial-temporal Gaussian process regression parameters 28
Appendix Table 7. Candidate covariates for covariate selection in network meta-regression 29
Author contributions 30
Managing the estimation or publications process 30
Writing the first draft of the manuscript 30
Primary responsibility for applying analytical methods to produce estimates 30
Primary responsibility for seeking, cataloguing, extracting, or cleaning data; designing or coding figures and tables 30
Providing data or critical feedback on data sources 30
Developing methods or computational machinery 30
Providing critical feedback on methods or results 30
Drafting the work or revising is critically for important intellectual content 30
Extracting, cleaning, or cataloging data; designing or coding figures and tables 31
Managing the overall research enterprise 31

Excluded datasets

We excluded ten databases on police-related violence that we found in our initial search from our analysis for a variety of issues, including incomplete case definitions of police violence, lack of detailed tabulations, and poor data quality. We chose to exclude the *Washington Post's* Fatal Force project, a commonly used open-source database, because it only included police shootings in its case definition of police violence. Notably, we also chose to exclude two governmental data-collection efforts on police violence, the National Violent Deaths Reporting System (NVDRS) and the Arrest-Related Deaths (ARD) program.

NVDRS is a surveillance system run by "frontline investigators including law enforcement, medical examiners, and coroners," who use death certificates, autopsy reports, and police reports to identify violent deaths.[1] Each state began its implementation of NVDRS at a different time, with the earliest states beginning in 2003 and the latest not yet reporting any data. Based on comparisons with two NVDRS-based studies covering 2005–2012 and 2009–2012, respectively, we calculate that NVDRS appears to report around 80-90% of deaths reported in Mapping Police Violence (MPV) and The Counted (TC).[2,3] One study found that NVDRS has approximately the same completeness as open-source databases when considering only police shootings.[4] Therefore the 10-20% gap between NVDRS and MPV/TC could be attributable to non-firearm police killings. We chose to exclude NVDRS from our analysis due to its apparent lack of coverage of non-firearm police killings, as well as its comparatively sparse spatial and temporal coverage. However, NVDRS does represent a significant improvement in the transparent reporting of fatal police violence by official sources in the USA.

ARD was a program run by the Bureau of Justice Statistics (BJS) from 2003 to 2014 that aimed to collect data on all deaths in the USA "during the process of arrest or while in the custody of state or local law enforcement."[5] ARD relied on State Reporting Coordinators to identify and report all arrest-related deaths in their state, but did not require a standard methodology for doing so. In 2014, ARD was discontinued when the BJS found that only 49% of police killings were identified in ARD from 2003 to 2009. We chose to exclude this data source since it showed similar under-reporting rates to that of NVSS.[5] Since then, BJS performed a study in 2015–2016 that showed that they were able to improve their coverage of police violence when they included open-source databases like Fatal Encounters (FE) and TC in their data collection.[6]

Biases in open-source data sources

The three open-source datasets used in this study have several important biases that were not accounted for in our network meta-regression approach (NMR), since they affect the gold-standard data, TC, as well as others. First, all open-source databases are limited by what events are publicized and what level of information is made available to the public from both law enforcement and eyewitnesses. For example, FE reports that most news media rely solely on the police department's narrative of events when reporting on police violence and do not seek out alternative accounts that may contradict them.[7] Another limitation of the Fatal Encounters source is that data collection was started in 2013 and occurred retrospectively for 2000-2012, potentially limiting the completeness of these early years. To address this, we analyzed the completeness of these years of FE and dropped those that we believe significantly underreport deaths (see "Completeness of Fatal Encounters" section below.)

Second, both MPV and TC use other open-source police databases as inputs in their data collection, including FE.[8,9] For example, MPV's sampling framework overlaps with that of Fatal Encounters, since MPV researchers compile their records based on Fatal Encounters, the US Police Shooting Database, and KilledbyPolice.net. They then subset these records to only those that fit their narrower definition of police killing, excluding any accidents or suicides captured in FE as well as any homicides perpetrated by civilians.[9] Since these datasets have correlated samples, we are likely underestimating the uncertainty of our estimates, and any police violence deaths not captured in our data sources are likely to be non-random.

Background on NVSS

The USA vital registration database is an exhaustive collection of deaths in the USA, with near 100% completeness when considering all causes of death.[10] We therefore expect that all police violence deaths that are not correctly coded to legal intervention in NVSS data are still present within the dataset, but coded to different diseases and injuries. Previous research has shown that for police violence deaths that are not correctly coded to legal intervention, the most common underlying cause of death that is listed instead is civilian assault.[11–13] Feldman and colleagues found that in the 2015 USA NVSS data, 86% of all police violence deaths not coded to legal intervention were coded to assault (X95-Y09). The second most common code range for unreported police violence deaths was

"events of undetermined intent or cause missing" (Y10-Y34, R99), at 4%.[13] The most likely cause of the high rate of miscoding to assault is that the medical examiner or coroner marks homicide as the manner of death, but fails to mention police involvement in the "describe how this injury occurred" section.[12]

Contestation of non-firearm deaths

An important potential source of bias that effects all data sources, including NVSS and the open-source databases, is the contestation of non-firearm deaths. In deaths involving Tasers, asphyxiation, and other non-firearm mechanisms, police statements and autopsy reports sometimes deny police culpability and claim that drug use, medical emergencies, and medically contested conditions like "excited delirium" are responsible for death.[14–16] This decreases the likelihood of police involvement being listed on the death certificate or police culpability being reported in media coverage of the death. This would potentially bias the completeness of both NVSS and open-source databases downwards. Non-firearm deaths comprise 8.0% of Fatal Encounters, 4.8% of MPV, 9.4% of TC, and 7.2% of NVSS.

Details on data standardisation

Age

We extracted and standardised the age, sex, USA state of death registration, year of death, and race/ethnicity of each decedent for all data sources. Across databases, age was generally presented in the raw data by year; we transformed these data into age groups using the bins established by the GBD 2020 study.[17] The age groups are: days: 0-6, 7-27; months: 1-5, 6-11, 12-23; years: 2-4, 5-9 , 10-14, 15-19, 20-24, 25-29, 30-34, 35-39, 40-44, 45-49, 50-54, 55-59, 60-64, 65-69, 70-74, 75-79, 80-84, 85-89, and 90-94. Binning age groups in this way allows for comparability across ages between data sources. For cases where there was less age detail than the standard bins, we used an age-sex-splitting algorithm for causes of death developed by the GBD study[17] to split these cases among our established bins.

Sex

Death certificates in the USA only allow a binary designation of sex and do not distinguish between sex assigned at birth and gender identity. Therefore, NVSS data conform to a male/female binary by design. In contrast, the open-source databases all record several decedents with their gender listed as transgender or non-binary. To standardise the data so that comparisons could be made, we chose to assign transgender and non-binary decedents in the open-source data to "sex unknown," which we then split into the male/female binary using the GBD age-sex-splitting algorithm for causes of death.[17]

Race and ethnicity

Data collection on the race and ethnicity of deceased individuals can be complicated and fraught with bias, as it is impossible for the decedent themselves to report the race and ethnicity with which they identify. NVSS relies on funeral directors to report the race/ethnicity of decedents on death certificates. The next of kin should in turn report this to the funeral director, but this does not always occur. Studies show that race/ethnicity reporting on death certificates is much poorer for American Indian and Alaskan Native decedents than other race/ethnicities.[18] Open-source databases rely on news reports and public records requests to identify race/ethnicity, which can also deviate from the decedent's self-identified race/ethnicity. MPV in particular makes an effort to improve the completeness and accuracy of its race/ethnicity variable by using police reports, criminal records, social media, and obituaries to find additional race/ethnicity information. Both NVSS and FE also attempt to impute race/ethnicity where it is missing; for example, NVSS attempts to impute Hispanic ethnicity based on place of birth, while FE uses surname and place of residence to impute race and ethnicity.[7,18]

Handling unknown race & ethnicity

For each data source, we reassigned deaths with unknown race/ethnicity to our standard race/ethnicity groupings proportionally based on the pattern of race/ethnicity among deaths with known race/ethnicity for each data source, year, and state. For data source/year/states with 0 deaths with known race/ethnicity, we pooled across year to obtain proportions. This method that assumes that a decedent's likelihood of being recorded with unknown race/ethnicity in a given dataset is independent of their true race/ethnicity. This is a questionable assumption based on established research on race/ethnicity measurement, so we attempted to ensure that that the proportion of deaths with unknown race/ethnicity was low enough for every data source and demographic to minimise the impact of this assumption[18] To this end, two data sources, NVSS and FE, required supplemental algorithms for the reassignment of unknown

race/ethnicity. An alternative method that could improve our results would be to backfill race/ethnicity between data sources for those data sources where records can be accurately linked and are permissible to link given the relevant laws and practices on individual privacy.

Unknown ethnicity in NVSS

NVSS has no police violence deaths with unknown race, but does have decedents with unknown ethnicity. Most state-years of NVSS have well below 10% ethnicity missingness, but each state has a distinct time series of 3 to 17 years starting from 1980 with > 50 % ethnicity missingness (Appendix Table 1). To assign race/ethnicity in these state-years, first we calculated the relative police violence mortality rate between Hispanic and non-Hispanic for each state (l), race (ρ), and sex (s) within the 5 years ($Y5$) immediately succeeding the time series with > 50% ethnicity missingness. Since we did not have population estimates separately by each race (white, Black, and other) for Hispanic people, we calculated these relative rates using the overall Hispanic population for all races:

$$\text{relative rate}_{l,\rho,s} = \frac{\text{police violence deaths}_{l,\rho,s,Y5,\text{Hispanic}} / \text{population}_{l,s,Y5,\text{Hispanic}}}{\text{police violence deaths}_{l,\rho,s,Y5,\text{non-Hispanic}} / \text{population}_{l,s,Y5,\text{non-Hispanic}}}$$

We then assumed that this relative rate would hold for all preceding years and applied these relative mortality rates to each year y with > 50% ethnicity missingness to split deaths with unknown race/ethnicity:

$$\text{fraction Hispanic}_{l,\rho,s,y} = \frac{\text{relative rate}_{l,\rho,s} \times \text{population}_{l,s,y,\text{Hispanic}}}{\text{population}_{l,s,y,\text{non-Hispanic}} + \text{relative rate}_{l,\rho,s} \times \text{population}_{l,s,y,\text{Hispanic}}}$$

By using population estimates that are not detailed by race, we assumed that the change in percent Hispanic population over time is the same for every race group. If the 5 years following a given time series had 0 police violence deaths for a given state, race, and sex, we progressively calculated 10, 20, and 40 year pools until a viable relative rate was found. A total of 3 deaths had no viable relative rate in even a 40-year pool, which we assigned to non-Hispanic.

Unknown race/ethnicity in Fatal Encounters

22% of all deaths in Fatal Encounters are missing race/ethnicity; however, the amount of missingness varies dramatically from year to year. Missingness is above 20% from 2005-2011 before lowering substantially from 2012-2017, and finally rising again in 2018-2019 (see Appendix Figure 1). The researchers who produce the Fatal Encounters say that the reliability of their race/ethnicity variable is best after 2013. In a prior analysis, Edwards et al found that known race/ethnicity distribution of Fatal Encounters diverges significantly from that of NVSS prior to 2013.[19] Based on the assumption that underreporting of police violence in NVSS is not affected by race/ethnicity, the researchers concluded that this divergence was an indication of biased known race/ethnicity. Any bias in the distribution of deaths with known race/ethnicity would be propagated by our method of proportionally redistributing unknown race/ethnicity.

To mitigate this problem, for decedents with unknown race/ethnicity, we used the imputed race/ethnicity provided by Fatal Encounters whenever the individual imputation probability was greater than 80%. Fatal Encounters uses the Bayesian Improved Surname Geocoding algorithm to obtain their imputations based on surname and place of residence.[8] This algorithm is the most accurate for people identified as Black and Asian and least for people identified as Indigenous.[20] We tested imputation probability cutoffs at every 10th percent (e.g. 0%, 10%, 20%, …) and chose the highest cutoff with which the percent of deaths with unknown race/ethnicity was reduced to less than 20% for all years from 2005-2019 (see Appendix Figure 1). After the inclusion of these imputed race/ethnicity values, we then redistributed all remaining deaths with unknown race/ethnicity according to the proportional method described above.

To validate the results of this approach, we replicated the analysis performed by Edwards et al, comparing the known race/ethnicity distribution of deaths in Fatal Encounters to that in NVSS. We found that the proportions of non-Hispanic white and non-Hispanic Black deaths in particular were more consistent between Fatal Encounters and NVSS after the inclusion of imputed race/ethnicity (see Appendix Figure 2). The proportions were still not completely consistent between Fatal Encounters and NVSS even after the inclusion of imputed race. However, the time trends of proportion across year were consistent between the two data sources for all race/ethnicity groups. There are observed, summary-level differences in underreporting in NVSS between racial/ethnic groups which may

explain these biases between NVSS and Fatal Encounters. Regardless, since this bias now consistent across year, our network meta-regression approach is capable of quantifying it and correcting it in the final results.

Standardizing race/ethnicity groups

Additionally, the race/ethnicity groups considered during data collection also vary between data sources. For example, NVSS classifies people of Middle Eastern and North African (MENA) descent as white during data collection; this practice does not accurately reflect the identities of all people from MENA.[21,22] The open-source databases do not make the distinction between race and ethnicity as NVSS does, forcing us to make some broad generalisations when attempting to standardise their race/ethnicity groups.

As discussed in the full text, for our primary analysis by state and four race/ethnicity groups, we chose to standardise all data sources to the race/ethnicity groups non-Hispanic Black, non-Hispanic white, non-Hispanic of other race, and Hispanic of any race. Non-Hispanic of other race includes people who are identified as Asian, Pacific Islander, Native Hawaiian, Indigenous, Native American, Alaskan Native, American Indian, Middle Eastern, North African, and Arab. For our secondary analysis at the national level with five race/ethnicity groups, we chose to standardise all data sources to the race ethnicity groups non-Hispanic Black, non-Hispanic white, non-Hispanic Indigenous, non-Hispanic of other race, and Hispanic of any race. For this secondary analysis, non-Hispanic of other race includes people who are identified as Asian, Pacific Islander, Native Hawaiian, Middle Eastern, North African, and Arab. Because none of the open-source databases reported decedents who identified as two or more races, we consider our standard categories to refer to only one identified race of each decedent. This assumption most likely does not reflect the true identities of decedents, but was necessary due to the lack of appropriate data. Appendix Table 4 shows the race/ethnicity categories in the raw data for each dataset and how we classified them to our standard race/ethnicity groups for both primary and secondary analyses.

There some key contradictions in our standardisation of NVSS that we address in our network-meta regression. We classified people of MENA descent as non-Hispanic, other race in the open-source databases, whereas NVSS includes MENA people as part of non-Hispanic white. Additionally, unlike the open-source databases, NVSS allows for the designation of more than one race per person in some years and states. The network meta-regression allows us to adjust the non-Hispanic white and non-Hispanic other categories in NVSS for any bias due to these alternative classifications of MENA and multiracial people to match the classifications that were used for the open-source databases.

Special handling of Fatal Encounters

Causes of death in Fatal Encounters

Given the fact that the FE database tracks deaths occurring during all police encounters, we assumed that any death in FE with cause listed as “Vehicle,” “Drug overdose,” “Undetermined,” “Medical emergency,” “Other,” or “Unknown” would be less likely to involve direct violence perpetrated by the police, and therefore excluded them. 22% of deaths from 2005–2019 were excluded from our analysis of the FE database for this reason. A major limitation of this assumption is that in cases where there are no eyewitnesses, even the open-source methodology relies on police and autopsy reports, which incorrectly over-emphasise the role of drugs and medical emergencies through medically contested conditions like “excited delirium,” which may be rooted in racial stereotypes.[14–16]

Completeness of Fatal Encounters

Edwards et al previously reported that the early years of Fatal Encounters likely undercount deaths, based on the assumption that underreporting in NVSS has been constant over time.[19] We followed the method used by Edwards et al to assess which years of Fatal Encounters are complete enough to include in our analysis. We normalised the total number of deaths in each year to the totals in 2000 separately for each data source (Appendix Figure 3). From this we observed that the normalised deaths reach a stable ratio between data sources in 2005, which continues for the rest of the time series. When the deaths in each data source are normalised to 2005, the normalised deaths are very consistent between Fatal Encounters and NVSS from 2005 onwards.

Based on this analysis, we believe that Fatal Encounters 2000-2004 undercount deaths and therefore excluded these data from all analyses. While it possible that the foundational assumption of this analysis is wrong and that the trends observed from 2000-2004 are a change in under-reporting levels in NVSS, we believe that this is unlikely for a number of reasons. First, underreporting in NVSS is remarkably consistent in all other years and we found no historical or policy-based reason why underreporting would have been lower in 2000-2004. Furthermore, Fatal

Encounters data collection for 2000-2004 occurred retroactively when the project was started in 2012, and computerised news reports are likely less abundant from this time.

Edwards et al reached a slightly different conclusion from their analysis of the completeness of Fatal Encounters, even though an identical method was used: they concluded that 2000-2007 of Fatal Encounters undercount deaths. There are several possible explanations for this disagreement. First, Edwards et al performed their analysis in 2019; since then, there have been revised releases of both NVSS and Fatal Encounters data. Second, we chose a different set of causes of death in Fatal Encounters to drop compared to Edwards et al. This may have affected the completeness of the remaining causes, since certain causes of death may be more likely to be reported by the news media than others.

Modelling

Estimating population

We generated direct and indirect comparisons for the NMR by taking the ratio between the cause-specific mortality rates for police violence in each USA state, year, and race/ethnicity group that any two datasets had in common, aggregating age and sex. To obtain these cause-specific mortality rates, we first estimated the population for each age group, sex, state, race/ethnicity group, and year from 1980 to 2020 for 50 states and the District of Columbia using bridged race/ethnicity population estimates from the NVSS[23] interpolated for the intercensal years. In a small number of strata, very low population counts, likely due to undercounts in the 1980 and 1990 censuses, led to implausible all-cause mortality rate estimates. In those few cases, we used a simple Gaussian process model to adjust the population values for 1980–1999.[24] Race/ethnicity-specific population estimates were then scaled to state-level population values from the GBD 2020 study, such that the sum of the four race/ethnicity populations for each state was equal to the state-level value.

Dealing with zeros

Since our implementation of NMR relies on a log transform of the ratio between mortality rates, data points with zero death counts for a given state/race/ethnicity/year are not usable. Excluding zeros entirely leads to bias in the model predictions; in particular, when dropping zeros, the NMR significantly underestimates the level of under-reporting in NVSS. To solve this problem, we offset every data point by a small amount prior to NMR, such that the total offset per state and year was one death, split proportionally across race/ethnicity at the state level.[17] After running the NMR and applying the resulting correction factors to each dataset, the deaths added to offset the data were subtracted off again to avoid inflating final estimates. Offsetting all data points ensures that, while the absolute counts are temporarily inflated, the ratios between them are not seriously affected on average. For all non-zero data points where this comparison is possible, the concordance correlation coefficient between the log ratios pre- and post-offset was 0.99. The average percent change in the ratios due to the offset was 2.4%, with a standard deviation of 9.7%. The concordance between the ratios pre- and post- offset decreases slightly as the pre-offset ratio approaches 0 or infinity (Appendix Figure 4). Offsetting increases the amount of data available to the regression and gives the regression a more accurate distribution of the raw data by enabling the inclusion of wider tails that represent state/race/ethnicity/years with 0 deaths (Appendix Figure 5).

Covariate selection

We based our list of candidate covariates on prior research on correlates of underreporting of police violence, our prior knowledge of the causes of underreporting, and the availability of data (Appendix Table 7). Transforms were tested for several covariates, notably log transforms for completeness and LDI to improve linearity and heteroscedasticity. All covariates were tested for collinearity. We then tested each covariate to see if its coefficient in the regression had a plausible direction and was statistically significant ($p\text{-value} < 0.05$). For the two categorical covariates, we ran one- and two-way ANOVA tests to determine significance. Only 3 covariates passed these criteria: percent of police violence deaths through the mechanism of firearm; state; and race. In particular, completeness, percent garbage, percent well-certified, drug, suicide, and homicide mortality rates, and LDI per capita by state/race/ethnicity were all insignificant, and LDI per capita by state and LDI per capita by state for non-Hispanic Black people were both insignificant and had implausible directions (negative).

Modelling assumptions

The NMR model specification assumes that under-reporting in NVSS varies by state and race/ethnicity, which is well-supported by the literature.[13] We assume that dataset-level biases of FE and MPV are constant over time, which is well supported by the data. An exception is 2000–2004 in FE, which show significantly fewer deaths than expected given constant under-reporting in NVSS. We believe this is more likely due to low completeness in FE rather than changing under-reporting in NVSS, and so we dropped FE 2000–2004 from all analyses.

We also assume that all dataset-level biases are constant across age and sex, since we aggregated these before generating between-dataset comparisons. This assumption may not always apply to children under 18, who in general have higher rates of under-reporting,[13] but, given that most police violence is committed against young and middle-aged adults, this assumption is reasonable. To test the assumption of constant underreporting across age group, we aggregated deaths from each data source across state, race/ethnicity, and sex and calculated the log deaths ratio between NVSS and each open-source database for a given year and age group. Aggregation was necessary in order to prevent 0 death counts which can bias the estimation. We limited the data to ages 20-54, since the vast majority of police violence deaths occur in these age groups, and ran a two-way ANOVA of log deaths ratio on age group and open-source database. We found there was no significant difference in log deaths ratio across the levels of age group (p-value = 0.327).

We performed an analogous ANOVA test for sex, in which we aggregated across age instead of sex and ran a two-way ANOVA of log deaths ratio on sex and open-source database. We found that there was a significant difference in log deaths ratio across the levels of sex (p-value = 2.12×10^{-4}). We then ran a linear regression of the log deaths ratio on sex and open-source database to quantify this effect. We found that the linear regression predicted an overall under-reporting rate for NVSS of 53% in males and 62% in females. However, given the low counts of deaths in females, we were unable to support the disaggregation of sex in our network meta-regression, so we made the simplifying assumption that underreporting is constant across sex. Future work could include address these limitations of our methods.

Estimating age and sex

As explained in the methods section, although all data sources included the age and sex of decedents, we chose to run all models on data aggregated across age and sex. We produced age and sex specific estimates by applying the GBD causes of death age-sex-splitting algorithm on our model predictions. We chose do this because we found that our modelling techniques were not robust enough to handle the small tabulation groups created when modelling by detailed age and sex. In particular, including detailed age and sex in models greatly increases the number of 0s that require offsetting, skewing the distribution of the log mortality rate ratios beyond what we judged to be appropriate. It also increases the stochasticity of the data and number of small data points in ST-GPR, which leads to poor fit and upward bias when the model is run with a log transform.

Raking in ST-GPR

ST-GPR runs on multiple levels of detail in location, race, and ethnicity. The most detailed set of data that ST-GPR is run on is most-detailed state/race/ethnicity group, as shown in the main text. ST-GPR also runs at the state level with no race/ethnicity detail, and finally at the national level with no race/ethnicity detail. We produce a separate stage 1 Poisson model for each level of detail using the same method described in the main test. After ST-GPR completes at each location level, we reconcile the results through a process known as raking. First, we scale the state-level estimates to the national level; then, we scale the most-detailed state/race/ethnicity estimates to the state level. We scale the more-detailed estimates to fit the less detailed because data with large sample sizes and fewer 0s are more stable in ST-GPR; therefore we trust these results more.

Calculating uncertainty

We calculated the uncertainty of our estimates using methods for network meta-regression and ST-GPR that are reported in detail elsewhere. [25,26] Briefly, we first calculated the sampling variance of each mortality rate in the data based on the assumption that police violence follows a Poisson distribution. We obtained a distribution of the coefficients of the network meta-regression by performing a parametric bootstrap that resampled the data and refit the optimization problem 1000 times. We calculated the variance of the correction factors from the NMR using this distribution. To obtain the uncertainty of the final ST-GPR estimate, we drew 1000 samples from multivariate normal distributions that include the sampling variance of the data, the variance of the NMR correction factors, and

the covariance of the Gaussian process itself. We performed all calculations on these 1000 samples and calculated the 2.5th and 97.5th percentiles across these samples to obtain the uncertainty interval for every estimate.

Uncertainty from sampling error, the network meta-regression, and ST-GPR are all included in our uncertainty intervals. The uncertainty associated with the following sources were not included in our uncertainty intervals: the population estimates that we used to calculate mortality rates; the algorithms we used to reassign deaths with unknown race/ethnicity; the correlated sampling frameworks of the open-source databases; and the secondary national/five race-ethnicity group network meta-regression.

Sensitivity analyses

To assess the impact of several of our modelling assumptions, we performed sensitivity analyses. We ran network meta-regressions (NMRs) to quantify the biases between data sources as described in the methods section of this paper, with the following changes:

1. Dropping the random effect on matching groups from the regression, to test its impact due to concerns that it may be collinear with some of the fixed effects in the model.
2. Dropping the time-variant covariate, percent of police violence deaths through firearms in NVSS, to test the impact of covariates on the time trend of the police violence estimates.
3. Dropping Fatal Encounters data from 2005-2012, in addition to 2000-2004, to assess the impact of these years of data. A previous study that used Fatal Encounters as a data source dropped these years.[19]

We then adjusted the data according to each NMR and compared the results of each regression using Lin's concordance correlation coefficient (CCC). The model without random effects had a CCC within 1e-15 of 1 when compared with our final results, indicating that our analysis is extremely robust to this random effect. The model without time-variant covariates had a CCC of 0.997 when compared to our final results. This means that given the universe of possible causal and explanatory factors of underreporting in NVSS that we considered in the analysis (Appendix Table 7), we predict that the rate of underreporting in NVSS has been mostly stable over time since 1980. The model without FE 2005-2012 had a CCC of 0.94 compared to our final results. Specifically, dropping FE 2005-2012 lead to higher estimates for NVSS, with an average increase of 63 deaths per year. The decision to drop or keep 2005-2012 FE data is a consequential one; however, based on our analyses of the completeness and quality of FE, we found no justification for rejecting these years of data. Since the final analysis contains all available data that met our quality standards, we trust its result above models that lack subsets of that data.

Incarceration

While the scope of this paper does not cover the relationship between incarceration, police violence and systemic racism, many other studies have established the inextricable links between these systems.[27,28] Policing and its associated violence is embedded in the larger American criminal justice system, which includes incarceration.[28] The criminal justice system is rooted in policies and systems that target Black communities and other people of color, including but not limited to: the school to prison pipeline, the war on drugs, discriminatory stop and frisk practices and segregation laws and policies.[29–32] These policies and practices disproportionately impact Black people and other people of color, contributing to both increased interactions with the police and more broadly with the criminal justice system.[31,33] For instance, Human Rights Watch reported in 2000 that while Black people constitute only 13% of all drug users, they represent 35% percent of arrests and 74% percent of those sent to prison.[34] The USA has seen a massive increase in the rate of in incarceration: raising by over 200% between 1980 and 1996,[35] to what is now referred to as "mass incarceration",[36] with US incarceration rates higher than any other country in the world.[37] Like police violence, mass incarceration affects Black and Hispanic communities at disproportionate rates compared to non-Hispanic white people.[38] Incarceration and police violence are higher for both non-Hispanic Black people and for Hispanic people of any race than for non-Hispanic white people, as illustrated in Appendix Figure 6. However, Hispanic people of any race have relatively low incarceration rates as compared to the high rate of police violence, while non-Hispanic Black people have high rates of both incarceration and police violence (Appendix Figure 6).

References

1 Centers for Disease Control and Prevention. National Violent Death Reporting System (NVDRS). https://www.cdc.gov/violenceprevention/datasources/nvdrs/index.html (accessed Jan 8, 2021).

2 DeGue S, Fowler KA, Calkins C. Deaths due to use of lethal force by law enforcement: findings from the national violent death reporting system, 17 U.S. states, 2009–2012. *American Journal of Preventive Medicine* 2016; **51**: S173–87.

3 Barber C, Azrael D, Cohen A, *et al.* Homicides by Police: Comparing Counts From the National Violent Death Reporting System, Vital Statistics, and Supplementary Homicide Reports. *Am J Public Health* 2016; **106**: 922–7.

4 Conner A, Azrael D, Lyons VH, Barber C, Miller M. Validating the National Violent Death Reporting System as a Source of Data on Fatal Shootings of Civilians by Law Enforcement Officers. *Am J Public Health* 2019; **109**: 578–84.

5 Bureau of Justice Statistics (BJS) - Arrest-Related Deaths. https://www.bjs.gov/index.cfm?tid=82&ty=tp (accessed Jan 6, 2021).

6 Banks D, Ruddle P, Kennedy E, Planty MG. Arrest-related deaths program redesign study, 2015-16: preliminary findings. U.S. Department of Justice: Office of Justice Programs, Bureau of Justice Statistics, 2016.

7 D. Brian Burghart. Fatal Encounters Database (United States). http://internal-ghdx.healthdata.org/record/fatal-encounters-database (accessed Jan 13, 2021).

8 Fatal Encounters – A step toward creating an impartial, comprehensive and searchable national database of people killed during interactions with police. https://fatalencounters.org/ (accessed Oct 7, 2020).

9 Mapping Police Violence. Mapping Police Violence. https://mappingpoliceviolence.org (accessed Sept 23, 2020).

10 Phillips DE, Lozano R, Naghavi M, *et al.* A composite metric for assessing data on mortality and causes of death: the vital statistics performance index. *Popul Health Metr* 2014; **12**: 14.

11 Loftin C, McDowall D, Xie M. Underreporting of Homicides by Police in the United States, 1976-2013. *Homicide Studies* 2017; **21**: 159–74.

12 Loftin C, Wiersema B, McDowall D, Dobrin A. Underreporting of Justifiable Homicides Committed by Police Officers in the United States, 1976–1998. *Am J Public Health* 2003; **93**: 1117–21.

13 Feldman JM, Gruskin S, Coull BA, Krieger N. Quantifying underreporting of law-enforcement-related deaths in United States vital statistics and news-media-based data sources: A capture–recapture analysis. *PLOS Medicine* 2017; **14**: e1002399.

14 Kodikara S, Cunningham K, Pollanen MS. “Excited delirium syndrome”: Is it a cause of death? *Legal Medicine* 2012; **14**: 252–4.

15 Lithwick D. Is “excited delirium” a diagnosis or a cover-up? Slate Magazine. 2015; published online June 11. https://slate.com/news-and-politics/2015/06/excited-delirium-deaths-in-police-custody-diagnosis-or-cover-up.html (accessed Oct 7, 2020).

16 Ranson D. Excited delirium syndrome: a political diagnosis? *J Law Med* 2012; **19**: 667–72.

17 GBD 2019 Diseases and Injuries Collaborators. Global burden of 369 diseases and injuries in 204 countries and territories, 1990–2019: a systematic analysis for the global burden of disease study 2019. *The Lancet* 2020; **396**: 1204–22.

18 Arias E, Schauman WS, Eschbach K, Sorlie PD, Backlund E. The validity of race and Hispanic origin reporting on death certificates in the United States. *Vital Health Stat 2* 2008; : 1–23.

19 Edwards F, Lee H, Esposito M. Risk of being killed by police use of force in the United States by age, race–ethnicity, and sex. *PNAS* 2019; **116**: 16793–8.

20 Elliott MN, Fremont A, Morrison PA, Pantoja P, Lurie N. A new method for estimating race/ethnicity and associated disparities where administrative records lack self-reported race/ethnicity. *Health Serv Res* 2008; **43**: 1722–36.

21 No Middle Eastern Or North African Category On 2020 Census, Bureau Says. NPR.org. https://www.npr.org/2018/01/29/581541111/no-middle-eastern-or-north-african-category-on-2020-census-bureau-says (accessed Dec 31, 2020).

22 United States Census Bureau. 2015 National Content Test: Race and Ethnicity Analysis Report. https://www.census.gov/programs-surveys/decennial-census/2020-census/planning-management/final-analysis/2015nct-race-ethnicity-analysis.html (accessed Jan 8, 2021).

23 National Center for Health Statistics (U.S.), editor. U.S. census 2000 population with bridged categories. Hyattsville, Md: National Center for Health Statistics, 2003.

24 Erickson C. GauPro: Gaussian Process Fitting. 2017 https://CRAN.R-project.org/package=GauPro (accessed Oct 15, 2020).

25 Appendix 1: Methods appendix to "Global burden of 87 risk factors in 204 countries and territories, 1990–2019: a systematic analysis for the Global Burden of Disease Study 2019". https://www.thelancet.com/cms/10.1016/S0140-6736(20)30752-2/attachment/54711c7c-216e-485e-9943-8c6e25648e1e/mmc1.pdf (accessed Jan 15, 2021).

26 Zheng P, Barber R, Sorensen R, Murray C, Aravkin A. Trimmed Constrained Mixed Effects Models: Formulations and Algorithms. *Journal of Computational and Graphical Statistics* 2021; **0**: 1–34.

27 The Racialization of Crime and Punishment: Criminal Justice, Color-Blind Racism, and the Political Economy of the Prison Industrial Complex - Rose M. Brewer, Nancy A. Heitzeg, 2008. https://journals.sagepub.com/doi/abs/10.1177/0002764207307745?casa_token=O2wuKB7-x4wAAAAA:vVXBvWum6ErmZKs0wGiG7JujuP81h7woi1fJbSCmM7KJvZJMIvM2BKnff9PZC1VjDnCxEWuWWe-q (accessed Jan 12, 2021).

28 Initiative PP. Visualizing the racial disparities in mass incarceration. https://www.prisonpolicy.org/blog/2020/07/27/disparities/ (accessed Jan 12, 2021).

29 Jimenez T. Racial prejudice predicts police militarization. 2020; published online Nov 30. DOI:10.31234/osf.io/7uhge.

30 Heitzeg NA. The School-to-Prison Pipeline: Education, Discipline, and Racialized Double Standards: Education, Discipline, and Racialized Double Standards. ABC-CLIO, 2016.

31 Stop-and-Frisk Practices. New York Civil Liberties Union. 2007; published online July 31. https://www.nyclu.org/en/issues/racial-justice/stop-and-frisk-practices (accessed Jan 12, 2021).

32 Archer DN. The New Housing Segregation: The Jim Crow Effects of Crime-Free Housing Ordinances. *Michigan Law Review*; **118**: 61.

33 Provine DM. Unequal under Law: Race in the War on Drugs. University of Chicago Press, 2008.

34 Decades of Disparity. Human Rights Watch. 2009; published online March 2. https://www.hrw.org/report/2009/03/02/decades-disparity/drug-arrests-and-race-united-states (accessed Jan 12, 2021).

35 National Research Council. The Growth of Incarceration in the United States: Exploring Causes and Consequences. Washington, D.C: The National Academies Press, 2014 DOI:10.17226/18613.

36 Mass Incarceration. obo. https://www.oxfordbibliographies.com/view/document/obo-9780195396607/obo-9780195396607-0033.xml (accessed Jan 12, 2021).

37 Initiative PP. States of Incarceration: The Global Context 2018. https://www.prisonpolicy.org/global/2018.html (accessed Jan 12, 2021).

38 The Color of Justice: Racial and Ethnic Disparity in State Prisons. The Sentencing Project. https://www.sentencingproject.org/publications/color-of-justice-racial-and-ethnic-disparity-in-state-prisons/ (accessed Jan 7, 2021).

39 Wang H, Abbas KM, Abbasifard M, *et al.* Global age-sex-specific fertility, mortality, healthy life expectancy (HALE), and population estimates in 204 countries and territories, 1950–2019: a comprehensive demographic analysis for the Global Burden of Disease Study 2019. *The Lancet* 2020; **396**: 1160–203.

40 James S, Gubbins P, Murray C, Gakidou E. Developing a comprehensive time series of GDP per capita for 210 countries from 1950 to 2015. *Popul Health Metr* 2012; **10**: 12.

41 Feldman JM, Gruskin S, Coull BA, Krieger N. Quantifying underreporting of law-enforcement-related deaths in United States vital statistics and news-media-based data sources: A capture–recapture analysis. *PLOS Medicine* 2017; **14**: e1002399.

Compliance with the Guidelines for Accurate and Transparent Health Estimates Reporting (GATHER)

Checklist of information that should be included in new reports of global health estimates

Item #	Checklist item	Reported on page #
**Objectives and funding**		
1	Define the indicator(s), populations (including age, sex, and geographic entities), and time period(s) for which estimates were made.	2
2	List the funding sources for the work.	3
**Data Inputs**		
For all data inputs from multiple sources that are synthesised as part of the study:		
3	Describe how the data were identified and how the data were accessed.	6-7
4	Specify the inclusion and exclusion criteria. Identify all ad-hoc exclusions.	6, Appendix p. 2
5	Provide information on all included data sources and their main characteristics. For each data source used, report reference information or contact name/institution, population represented, data collection method, year(s) of data collection, sex and age range, diagnostic criteria or measurement method, and sample size, as relevant.	6-7, Appendix p. 3-4 and Table 1
6	Identify and describe any categories of input data that have potentially important biases (e.g., based on characteristics listed in item 5).	8, Appendix p. 2
For data inputs that contribute to the analysis but were not synthesised as part of the study:		
7	Describe and give sources for any other data inputs.	n/a
For all data inputs:		
8	Provide all data inputs in a file format from which data can be efficiently extracted (e.g., a spreadsheet rather than a PDF), including all relevant meta-data listed in item 5. For any data inputs that cannot be shared because of ethical or legal reasons, such as third-party ownership, provide a contact name or the name of the institution that retains the right to the data.	17
**Data analysis**		
9	Provide a conceptual overview of the data analysis method. A diagram may be helpful.	8, Figure 1
10	Provide a detailed description of all steps of the analysis, including mathematical formulae. This description should cover, as relevant, data cleaning, data pre-processing, data adjustments and weighting of data sources, and mathematical or statistical model(s).	8
11	Describe how candidate models were evaluated and how the final model(s) were selected.	8
12	Provide the results of an evaluation of model performance, if done, as well as the results of any relevant sensitivity analysis.	Appendix p. 4
13	Describe methods for calculating uncertainty of the estimates. State which sources of uncertainty were, and were not, accounted for in the uncertainty analysis.	Appendix p. 5
14	State how analytic or statistical source code used to generate estimates can be accessed.	Available through GHDx link (upon publication)
**Results and Discussion**		
15	Provide published estimates in a file format from which data can be efficiently extracted.	N/A
16	Report a quantitative measure of the uncertainty of the estimates (e.g. uncertainty intervals).	10-12
17	Interpret results in light of existing evidence. If updating a previous set of estimates, describe the reasons for changes in estimates.	13-14
18	Discuss limitations of the estimates. Include a discussion of any modelling assumptions or data limitations that affect interpretation of the estimates.	15

Supplemental figures

Appendix Figure 1. Distribution of specified and unspecified race/ethnicity in Fatal Encounters, before and after including imputed race/ethnicity

Proportion of deaths with unknown race/ethnicity, before and after including imputed race/ethnicity values with > 80% imputation probability.

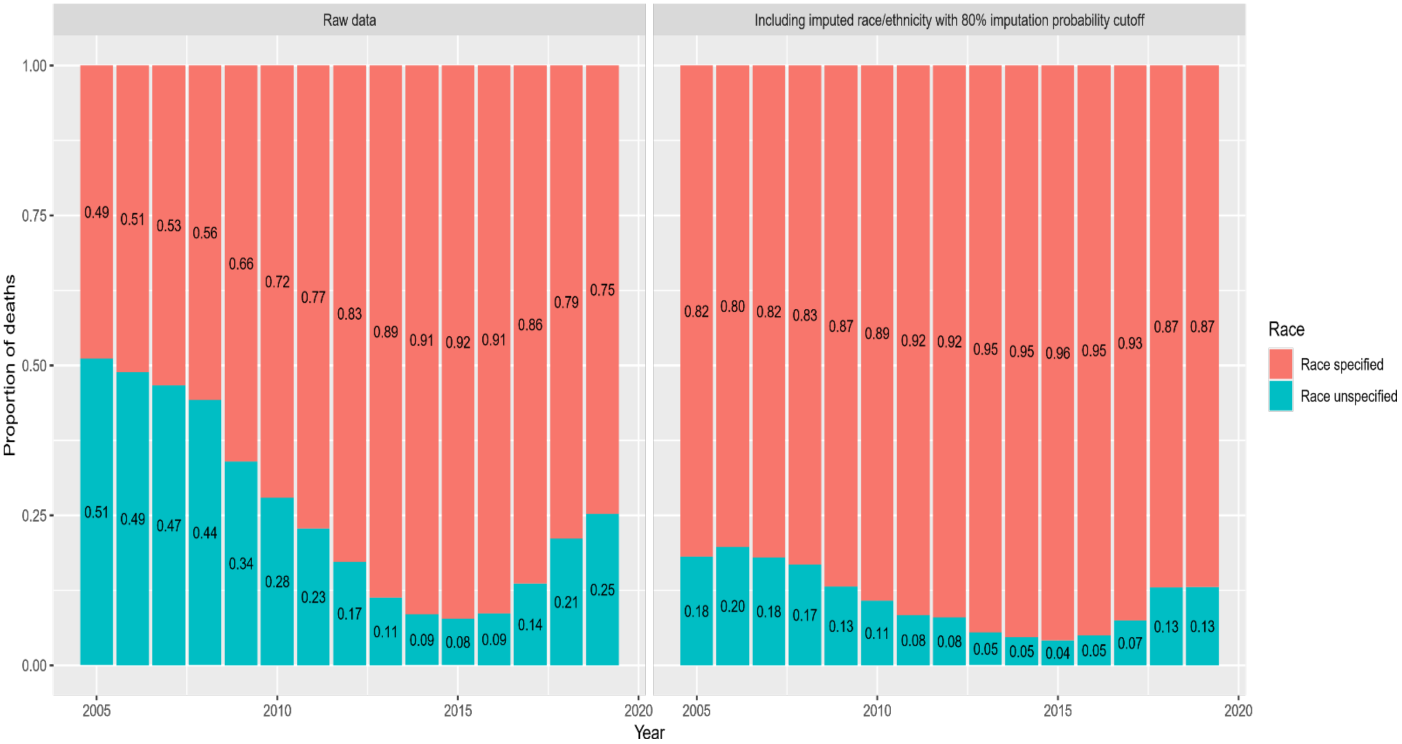

Appendix Figure 2. Race/ethnicity composition of Fatal Encounters and NVSS, before and after including imputed race/ethnicity in Fatal Encounters

Proportion of deaths with known race/ethnicity in each race ethnicity group, NVSS vs Fatal Encounters, before and after including imputed race/ethnicity values with > 80% imputation probability.

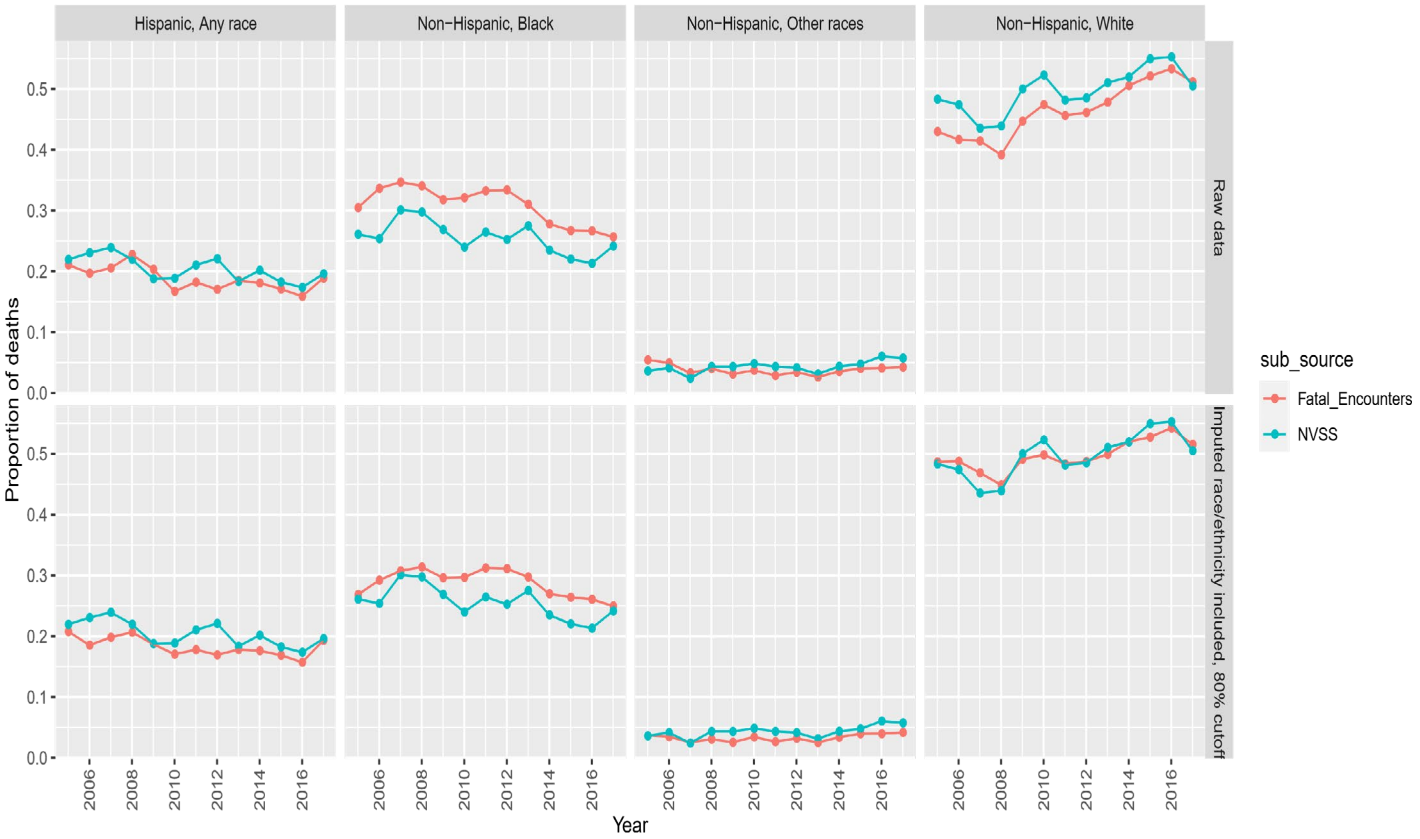

Appendix Figure 3. Normalised deaths in Fatal Encounters and NVSS

Deaths for each data source are normalised to 1 for a common year, making their time trends relative to this year comparable between data sources.

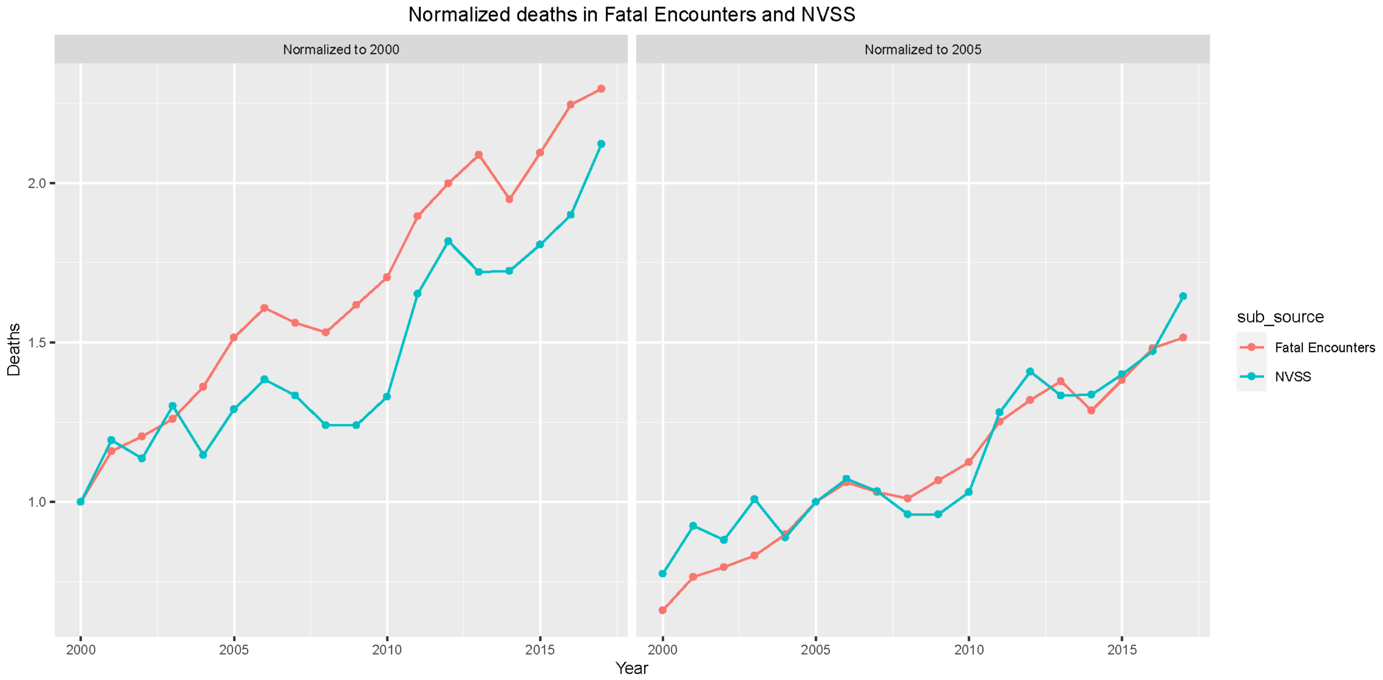

Appendix Figure 4. Ratios of mortality rates for all non-zero data points, pre- and post-offset

Each data point represents the ratio of the mortality rates between two data sources for a given state, race, ethnicity, and year. This ratio can only be calculated and log-transformed for data points where both data sources report non-zero deaths, so only these ratios are shown. The line of perfect concordance between pre- and post-offset is shown in blue.

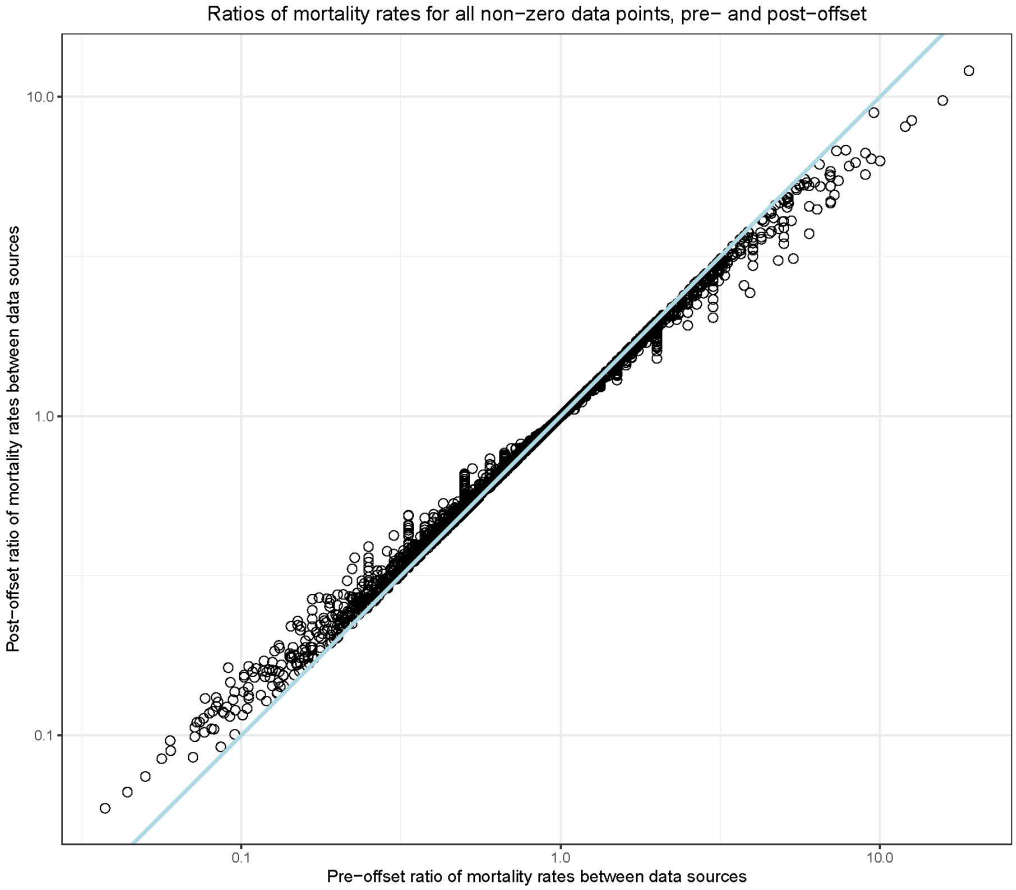

Appendix Figure 5. Distributions of ratios of mortality rates, pre- and post-offset

Each data point represents the ratio of the mortality rates between two data sources for a given state, race, ethnicity, and year.

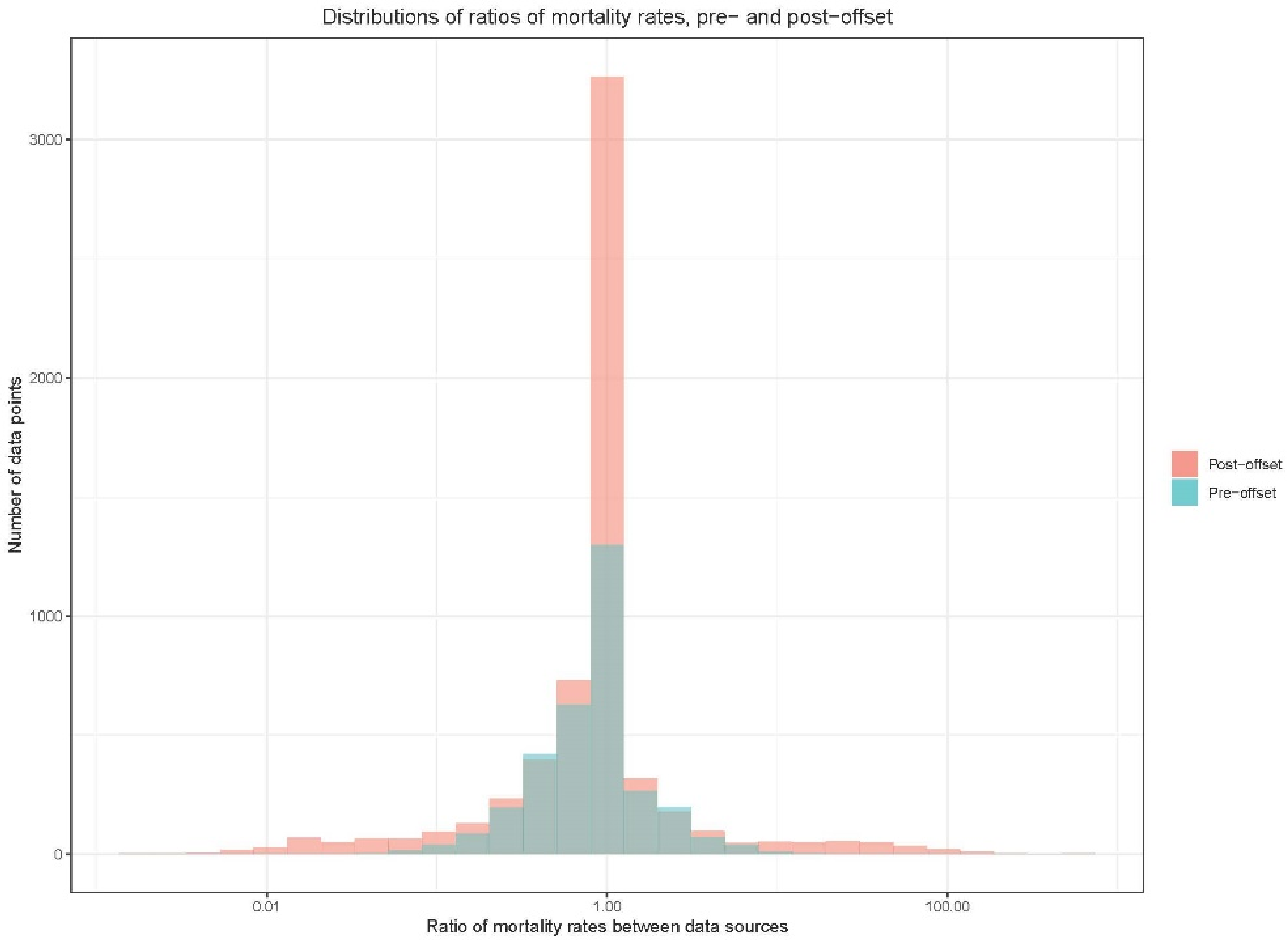

Appendix Figure 6. Police violence and incarceration rates per 100,000 by state, race, ethnicity, and year, 1980-2018

Each data point is a state/race/ethnicity/year, with only state-years with a population of 700,000 or greater included to avoid the stochastic effects. Police violence rates come from our modelled estimate, while the incarceration rates were extracted from the National Prisoner Statistics database collected by the US Bureau of Justice Statistics. Only prisoners incarcerated in long-term confinement under state jurisdiction are included in these rates. Prisoners incarcerated under federal jurisdiction and prisoners held in local jails not under state jurisdiction are not included. The line is the linear regression of police violence rate on incarceration rate and the shaded area is the 95% uncertainty interval of the regression.

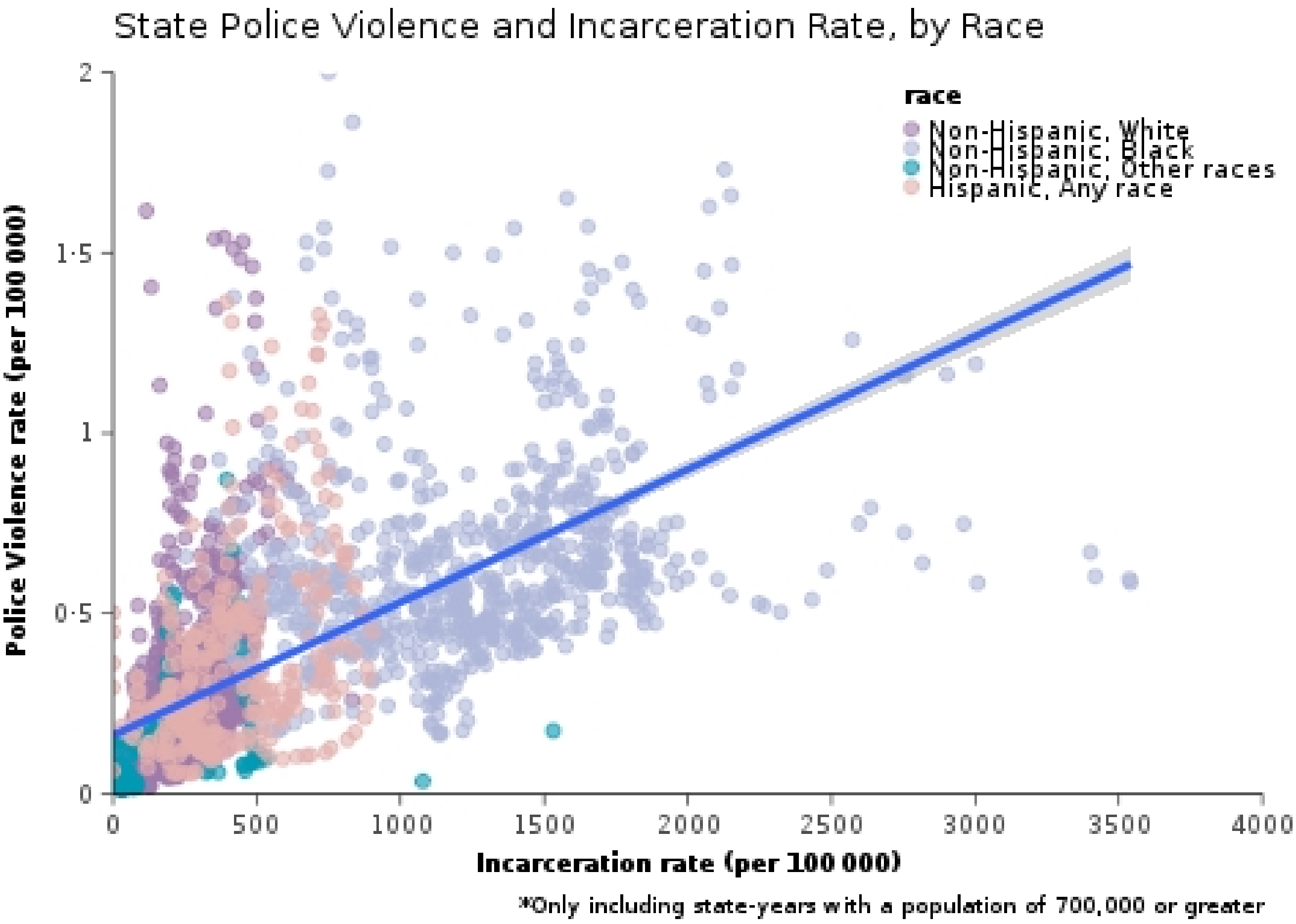

Appendix Figure 7: Methods for the estimation of police violence in the USA by state, race, and ethnicity, 1980-2019

The network meta-regression is represented in a network graph, where each data source is a node and each connection represents the type of comparison and number of years of data that inform that comparison. The inclusion of indirect comparisons adds 25 additional years of comparisons to the model.

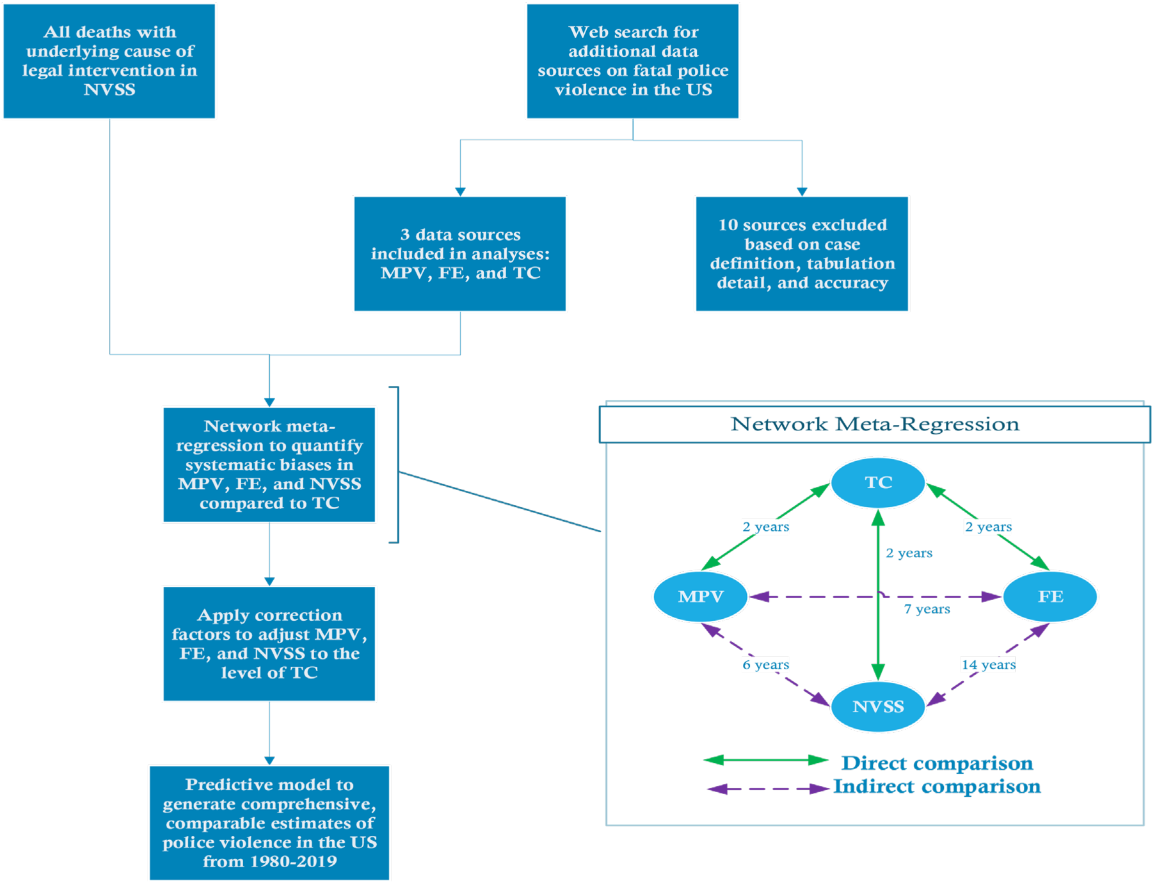

Supplemental Tables

Appendix Table 1. Percent of deaths missing ethnicity in NVSS by state and year, 1980-1999

Percent of deaths missing Hispanic ethnicity by state-year in NVSS from 1980-1999, including deaths due to all causes (not limited to police violence).

State	1980	1981	1982	1983	1984	1985	1986	1987	1988	1989	1990	1991	1992	1993	1994	1995	1996	1997	1998	1999
AK	100%	100%	100%	100%	98%	98%	98%	98%	93%	3%	0%	1%	1%	0%	0%	0%	0%	0%	0%	0%
AL	100%	100%	100%	100%	97%	97%	97%	97%	2%	1%	1%	0%	0%	0%	0%	0%	0%	0%	0%	0%
AR	100%	100%	100%	100%	26%	9%	4%	3%	3%	1%	1%	0%	0%	0%	0%	0%	0%	0%	0%	0%
AZ	100%	100%	100%	100%	1%	1%	1%	2%	1%	0%	0%	0%	0%	0%	0%	1%	0%	0%	0%	1%
CA	100%	100%	100%	100%	5%	2%	2%	2%	1%	1%	0%	0%	0%	0%	0%	0%	0%	0%	0%	0%
CO	100%	100%	100%	100%	6%	5%	5%	5%	4%	0%	0%	0%	0%	0%	0%	0%	0%	0%	0%	0%
CT	100%	100%	100%	100%	99%	99%	99%	99%	98%	63%	20%	6%	7%	2%	0%	0%	0%	1%	1%	1%
DC	100%	100%	100%	100%	93%	11%	13%	14%	16%	3%	1%	1%	0%	0%	0%	0%	0%	0%	1%	1%
DE	100%	100%	100%	100%	99%	99%	99%	99%	99%	1%	0%	0%	0%	0%	0%	0%	0%	0%	0%	1%
FL	100%	100%	100%	100%	98%	98%	98%	98%	97%	0%	0%	0%	0%	0%	0%	0%	0%	0%	0%	0%
GA	100%	100%	100%	100%	3%	3%	3%	3%	2%	1%	1%	1%	0%	0%	1%	0%	0%	0%	0%	0%
HI	100%	100%	100%	100%	1%	1%	1%	0%	0%	0%	0%	0%	0%	0%	0%	0%	0%	0%	0%	0%
IA	100%	100%	100%	100%	97%	97%	97%	97%	97%	0%	0%	0%	0%	0%	0%	0%	0%	0%	0%	0%
ID	100%	100%	100%	100%	97%	96%	96%	96%	92%	0%	0%	0%	0%	0%	0%	0%	0%	0%	0%	0%
IL	100%	100%	100%	100%	8%	8%	7%	8%	8%	1%	1%	1%	1%	1%	1%	1%	1%	1%	1%	0%
IN	100%	100%	100%	100%	3%	3%	3%	3%	1%	1%	1%	0%	0%	0%	0%	0%	0%	0%	0%	0%
KS	100%	100%	100%	100%	9%	9%	8%	8%	7%	3%	3%	3%	2%	2%	2%	2%	2%	1%	1%	1%
KY	100%	100%	100%	100%	97%	97%	96%	96%	2%	1%	1%	1%	0%	0%	0%	0%	0%	0%	0%	0%
LA	100%	100%	100%	100%	98%	98%	98%	98%	98%	98%	98%	0%	0%	0%	0%	0%	0%	0%	0%	0%
MA	100%	100%	100%	100%	99%	99%	99%	99%	99%	1%	1%	1%	0%	0%	0%	0%	0%	0%	0%	0%
MD	100%	100%	100%	100%	99%	95%	95%	95%	95%	17%	2%	2%	3%	2%	0%	0%	0%	0%	0%	0%
ME	100%	100%	100%	100%	23%	24%	23%	23%	18%	9%	7%	6%	5%	3%	4%	3%	3%	2%	1%	1%
MI	100%	100%	100%	100%	98%	98%	98%	99%	98%	2%	1%	1%	1%	1%	1%	1%	1%	1%	1%	1%
MN	100%	100%	100%	100%	98%	98%	98%	98%	97%	0%	0%	0%	0%	0%	0%	0%	1%	1%	1%	1%
MO	100%	100%	100%	100%	97%	97%	97%	97%	97%	0%	0%	0%	0%	0%	0%	0%	0%	0%	0%	0%
MS	100%	100%	100%	100%	4%	4%	3%	3%	2%	1%	1%	0%	0%	0%	0%	0%	0%	0%	0%	0%
MT	100%	100%	100%	100%	97%	97%	97%	97%	6%	3%	2%	1%	1%	1%	1%	1%	0%	0%	1%	0%
NC	100%	100%	100%	100%	99%	99%	99%	99%	3%	0%	0%	0%	0%	0%	0%	0%	0%	0%	0%	0%
ND	100%	100%	100%	100%	12%	11%	10%	11%	10%	3%	3%	2%	2%	3%	3%	3%	2%	3%	3%	3%
NE	100%	100%	100%	100%	10%	9%	9%	9%	8%	1%	1%	1%	1%	1%	1%	1%	1%	1%	0%	0%
NH	100%	100%	100%	100%	99%	99%	99%	99%	98%	90%	90%	90%	90%	7%	4%	4%	2%	2%	3%	2%
NJ	100%	100%	100%	100%	24%	22%	19%	18%	17%	1%	1%	1%	1%	1%	0%	2%	0%	0%	0%	0%
NM	100%	100%	100%	100%	1%	1%	1%	1%	0%	0%	0%	0%	0%	0%	0%	0%	0%	0%	0%	0%
NV	100%	100%	100%	100%	31%	34%	36%	38%	31%	0%	0%	2%	3%	0%	2%	2%	2%	1%	0%	0%
NY	100%	100%	100%	100%	7%	9%	10%	11%	8%	4%	8%	8%	10%	10%	2%	2%	1%	1%	1%	2%
OH	100%	100%	100%	100%	10%	10%	10%	9%	8%	1%	0%	0%	0%	0%	0%	0%	1%	1%	1%	0%
OK	100%	100%	100%	100%	96%	96%	96%	95%	95%	95%	95%	95%	95%	95%	95%	95%	95%	0%	0%	0%

OR	100%	100%	100%	100%	99%	99%	99%	99%	1%	0%	0%	0%	0%	0%	0%	0%	0%	0%	0%	0%
PA	100%	100%	100%	100%	99%	99%	99%	99%	98%	0%	0%	0%	0%	0%	0%	0%	0%	0%	0%	0%
RI	100%	100%	100%	100%	99%	100%	100%	100%	13%	1%	1%	1%	1%	1%	1%	1%	1%	1%	1%	1%
SC	100%	100%	100%	100%	97%	97%	97%	97%	95%	0%	0%	0%	0%	0%	0%	0%	0%	0%	0%	0%
SD	100%	100%	100%	100%	96%	97%	96%	96%	96%	0%	0%	0%	0%	0%	0%	0%	0%	0%	0%	0%
TN	100%	100%	100%	100%	35%	19%	14%	14%	12%	1%	1%	1%	1%	0%	0%	0%	0%	0%	0%	0%
TX	100%	100%	100%	100%	1%	1%	1%	1%	1%	1%	1%	1%	0%	0%	0%	0%	0%	0%	0%	0%
UT	100%	100%	100%	100%	2%	1%	1%	1%	1%	0%	0%	0%	0%	0%	0%	0%	0%	0%	0%	0%
VA	100%	100%	100%	100%	99%	98%	98%	98%	97%	28%	3%	2%	1%	1%	0%	0%	0%	1%	1%	1%
VT	100%	100%	100%	100%	99%	99%	99%	99%	99%	12%	7%	6%	7%	1%	0%	0%	0%	0%	0%	0%
WA	100%	100%	100%	100%	99%	99%	99%	99%	2%	0%	0%	0%	0%	0%	0%	0%	0%	0%	0%	0%
WI	100%	100%	100%	100%	99%	99%	99%	99%	99%	0%	0%	0%	0%	0%	0%	0%	0%	0%	0%	0%
WV	100%	100%	100%	100%	98%	97%	97%	98%	96%	1%	0%	0%	0%	0%	0%	0%	0%	0%	0%	0%
WY	100%	100%	100%	100%	9%	8%	8%	7%	6%	0%	0%	0%	0%	0%	0%	0%	0%	0%	0%	0%

Appendix Table 2. Legal intervention ICD 10 codes used to identify police violence in NVSS

Six-digit ICD codes for legal intervention show the identity of the person injured (police or civilian), but in practice codes with this level of detail are not used by NVSS.

**Y35**	**Legal intervention**
Y35.0	Legal intervention involving firearm discharge
Y35.00	Legal intervention involving unspecified firearm discharge
Y35.001	Legal intervention involving unspecified firearm discharge, law enforcement official injured, sequela
Y35.002	Legal intervention involving unspecified firearm discharge, bystander injured, sequela
Y35.003	Legal intervention involving unspecified firearm discharge, suspect injured, sequela
Y35.01	Legal intervention involving injury by machine gun
Y35.011	Legal intervention involving injury by machine gun, law enforcement official injured, sequela
Y35.012	Legal intervention involving injury by machine gun, bystander injured, sequela
Y35.013	Legal intervention involving injury by machine gun, suspect injured, sequela
Y35.02	Legal intervention involving injury by handgun
Y35.021	Legal intervention involving injury by handgun, law enforcement official injured, sequela
Y35.022	Legal intervention involving injury by handgun, bystander injured, sequela
Y35.023	Legal intervention involving injury by handgun, suspect injured, sequela
Y35.03	Legal intervention involving injury by rifle pellet
Y35.031	Legal intervention involving injury by rifle pellet, law enforcement official injured, sequela
Y35.032	Legal intervention involving injury by rifle pellet, bystander injured, sequela
Y35.033	Legal intervention involving injury by rifle pellet, suspect injured, sequela
Y35.04	Legal intervention involving injury by rubber bullet
Y35.041	Legal intervention involving injury by rubber bullet, law enforcement official injured, sequela
Y35.042	Legal intervention involving injury by rubber bullet, bystander injured, sequela
Y35.043	Legal intervention involving injury by rubber bullet, suspect injured, sequela
Y35.09	Legal intervention involving other firearm discharge
Y35.091	Legal intervention involving other firearm discharge, law enforcement official injured, sequela
Y35.092	Legal intervention involving other firearm discharge, bystander injured, sequela
Y35.093	Legal intervention involving other firearm discharge, suspect injured, sequela
Y35.1	Legal intervention involving explosives
Y35.10	Legal intervention involving unspecified explosives
Y35.101	Legal intervention involving unspecified explosives, law enforcement official injured, sequela
Y35.102	Legal intervention involving unspecified explosives, bystander injured, sequela
Y35.103	Legal intervention involving unspecified explosives, suspect injured, sequela
Y35.11	Legal intervention involving injury by dynamite
Y35.111	Legal intervention involving injury by dynamite, law enforcement official injured, sequela
Y35.112	Legal intervention involving injury by dynamite, bystander injured, sequela
Y35.113	Legal intervention involving injury by dynamite, suspect injured, sequela
Y35.12	Legal intervention involving injury by explosive shell
Y35.121	Legal intervention involving injury by explosive shell, law enforcement official injured, sequela
Y35.122	Legal intervention involving injury by explosive shell, bystander injured, sequela
Y35.123	Legal intervention involving injury by explosive shell, suspect injured, sequela
Y35.19	Legal intervention involving other explosives
Y35.191	Legal intervention involving other explosives, law enforcement official injured, sequela
Y35.192	Legal intervention involving other explosives, bystander injured, sequela
Y35.193	Legal intervention involving other explosives, suspect injured, sequela
Y35.2	Legal intervention involving gas

Y35.20	Legal intervention involving unspecified gas
Y35.201	Legal intervention involving unspecified gas, law enforcement official injured, sequela
Y35.202	Legal intervention involving unspecified gas, bystander injured, sequela
Y35.203	Legal intervention involving unspecified gas, suspect injured, sequela
Y35.21	Legal intervention involving injury by tear gas
Y35.211	Legal intervention involving injury by tear gas, law enforcement official injured, sequela
Y35.212	Legal intervention involving injury by tear gas, bystander injured, sequela
Y35.213	Legal intervention involving injury by tear gas, suspect injured, sequela
Y35.29	Legal intervention involving other gas
Y35.291	Legal intervention involving other gas, law enforcement official injured, sequela
Y35.292	Legal intervention involving other gas, bystander injured, sequela
Y35.293	Legal intervention involving other gas, suspect injured, sequela
Y35.3	Legal intervention involving blunt objects
Y35.30	Legal intervention involving unspecified blunt objects
Y35.301	Legal intervention involving unspecified blunt objects, law enforcement official injured, sequela
Y35.302	Legal intervention involving unspecified blunt objects, bystander injured, sequela
Y35.303	Legal intervention involving unspecified blunt objects, suspect injured, sequela
Y35.31	Legal intervention involving baton
Y35.311	Legal intervention involving baton, law enforcement official injured, sequela
Y35.312	Legal intervention involving baton, bystander injured, sequela
Y35.313	Legal intervention involving baton, suspect injured, sequela
Y35.39	Legal intervention involving other blunt objects
Y35.391	Legal intervention involving other blunt objects, law enforcement official injured, sequela
Y35.392	Legal intervention involving other blunt objects, bystander injured, sequela
Y35.393	Legal intervention involving other blunt objects, suspect injured, sequela
Y35.4	Legal intervention involving sharp objects
Y35.40	Legal intervention involving unspecified sharp objects
Y35.401	Legal intervention involving unspecified sharp objects, law enforcement official injured, sequela
Y35.402	Legal intervention involving unspecified sharp objects, bystander injured, sequela
Y35.403	Legal intervention involving unspecified sharp objects, suspect injured, sequela
Y35.41	Legal intervention involving bayonet
Y35.411	Legal intervention involving bayonet, law enforcement official injured, sequela
Y35.412	Legal intervention involving bayonet, bystander injured, sequela
Y35.413	Legal intervention involving bayonet, suspect injured, sequela
Y35.49	Legal intervention involving other sharp objects
Y35.491	Legal intervention involving other sharp objects, law enforcement official injured, sequela
Y35.492	Legal intervention involving other sharp objects, bystander injured, sequela
Y35.493	Legal intervention involving other sharp objects, suspect injured, sequela
Y35.6	Legal intervention involving other specified means
Y35.7	Legal intervention, means unspecified
Y35.8	Legal intervention involving other specified means
Y35.81	Legal intervention involving manhandling
Y35.811	Legal intervention involving manhandling, law enforcement official injured, sequela
Y35.812	Legal intervention involving manhandling, bystander injured, sequela
Y35.813	Legal intervention involving manhandling, suspect injured, sequela
Y35.89	Legal intervention involving other specified means
Y35.891	Legal intervention involving other specified means, law enforcement official injured, sequela
Y35.892	Legal intervention involving other specified means, bystander injured, sequela

Y35.893	Legal intervention involving other specified means, suspect injured, sequela
Y35.9	Legal intervention, means unspecified
Y35.91	Legal intervention, means unspecified, law enforcement official injured, sequela
Y35.92	Legal intervention, means unspecified, bystander injured, sequela
Y35.93	Legal intervention, means unspecified, suspect injured, sequela
Y89.0	Sequelae of legal intervention

Appendix Table 3. Legal intervention ICD 9 codes used to identify police violence in NVSS

E970	Injury due to legal intervention by firearms
E971	Injury due to legal intervention by explosives
E972	Injury due to legal intervention by gas
E973	Injury due to legal intervention by blunt object
E974	Injury due to legal intervention by cutting and piercing instrument
E975	Injury due to legal intervention by other specified means
E976	Injury due to legal intervention by unspecified means
E977	Late effects of injuries due to legal intervention

Appendix Table 4. Race/ethnicity group standardisation across data sources

All race/ethnicities categories reported in each data source and the standardised group to which we mapped it.

Data source	Race/ethnicity category in source	Standardised group	
		Primary analysis	Secondary analysis
Fatal Encounters	European-American/White	Non-Hispanic white	Non-Hispanic white
	African-American/Black	Non-Hispanic Black	Non-Hispanic Black
	Hispanic/Latino	Hispanic of any race	Hispanic of any race
	Native American/Alaskan	Non-Hispanic of other race	Non-Hispanic Indigenous
	Asian/Pacific Islander	Non-Hispanic of other race	Non-Hispanic of other race
	Middle Eastern	Non-Hispanic of other race	Non-Hispanic of other race
Mapping Police Violence	White	Non-Hispanic white	Non-Hispanic white
	Black	Non-Hispanic Black	Non-Hispanic Black
	Hispanic	Hispanic of any race	Hispanic of any race
	Pacific Islander	Non-Hispanic of other race	Non-Hispanic of other race
	Asian	Non-Hispanic of other race	Non-Hispanic of other race
	Native American	Non-Hispanic of other race	Non-Hispanic Indigenous
The Counted	White	Non-Hispanic white	Non-Hispanic white
	Black	Non-Hispanic Black	Non-Hispanic Black
	Hispanic/Latino	Hispanic of any race	Hispanic of any race
	Native American	Non-Hispanic of other race	Non-Hispanic Indigenous
	Asian/Pacific Islander	Non-Hispanic of other race	Non-Hispanic of other race
	Arab-American	Non-Hispanic of other race	Non-Hispanic of other race
	Other	Non-Hispanic of other race	Non-Hispanic of other race
NVSS race groups – *standardised group is overridden to Hispanic of any race when these appear with a Hispanic ethnicity*	Other Asian or Pacific Islander	Non-Hispanic of other race	Non-Hispanic of other race
	White	Non-Hispanic white	Non-Hispanic white
	Black	Non-Hispanic Black	Non-Hispanic Black
	American Indian incl. Aleuts & Eskimos	Non-Hispanic of other race	Non-Hispanic Indigenous
	Chinese	Non-Hispanic of other race	Non-Hispanic of other race
	Japanese	Non-Hispanic of other race	Non-Hispanic of other race
	Hawaiian incl. Part-Hawaiian	Non-Hispanic of other race	Non-Hispanic of other race
	All other races	Non-Hispanic of other race	Non-Hispanic of other race
	Filipino	Non-Hispanic of other race	Non-Hispanic of other race
	Other Races	Non-Hispanic of other race	Non-Hispanic of other race
	Guamanian	Non-Hispanic of other race	Non-Hispanic of other race

	Asian Indian	Non-Hispanic of other race	Non-Hispanic of other race
	Korean	Non-Hispanic of other race	Non-Hispanic of other race
	Samoan	Non-Hispanic of other race	Non-Hispanic of other race
	Vietnamese	Non-Hispanic of other race	Non-Hispanic of other race
	American Indian or Alaskan Native (AIAN)	Non-Hispanic of other race	Non-Hispanic Indigenous
	Hawaiian	Non-Hispanic of other race	Non-Hispanic of other race
	Other or Multiple Asian	Non-Hispanic of other race	Non-Hispanic of other race
	Other or Multiple Pacific Islander	Non-Hispanic of other race	Non-Hispanic of other race
	Specific Multiple Race Combinations	Non-Hispanic of other race	Non-Hispanic of other race
NVSS ethnicities – *standardised group is determined by race when not Hispanic*	Non-Spanish	Non-Hispanic	Non-Hispanic
	Mexican	Hispanic of any race	Hispanic of any race
	Puerto Rican	Hispanic of any race	Hispanic of any race
	Cuban	Hispanic of any race	Hispanic of any race
	Central or South American	Hispanic of any race	Hispanic of any race
	Other or unknown Spanish	Hispanic of any race	Hispanic of any race
	Other European, African, and Asian ethnicities	Non-Hispanic	Non-Hispanic
	Other or unknown Hispanic	Hispanic of any race	Hispanic of any race
	Non-Hispanic white	Non-Hispanic	Non-Hispanic
	Non-Hispanic Black	Non-Hispanic	Non-Hispanic
	Non-Hispanic other races	Non-Hispanic	Non-Hispanic

Appendix Table 5. Causes of death in Fatal Encounters, 2005–2019

Vehicle, medical emergency, drug overdose, undetermined, and other were excluded from analysis.

Cause of death	Deaths	Percentage of deaths
Gunshot	16046	71.4%
Vehicle*	4468	19.9%
Tasered	826	3.7%
Medical emergency*	310	1.4%
Asphyxiated/restrained	182	0.8%
Drug overdose*	146	0.7%
Drowned	125	0.6%
Beaten/bludgeoned with instrument	119	0.5%
Undetermined*	73	0.3%
Fell from a height	56	0.2%
Stabbed	33	0.1%
Other*	28	0.1%
Chemical agent/pepper spray	26	0.1%
Burned/smoke inhalation	22	0.1%
**Total**	22460	100.0%

**Excluded from analysis*

Appendix Table 6. Spatial-temporal Gaussian process regression parameters

Hyper-parameters of ST-GPR used to produce final estimates of police violence mortality in the USA by state, race, and ethnicity.

Parameter	Value
λ (time adjustment)	0.4
ω (age adjustment)	1.0
ζ (space adjustment)	0.001
l (temporal length scale)	5
ν (degree of differentiability)	2

Appendix Table 7. Candidate covariates for covariate selection in network meta-regression

Candidate covariates for covariate selection

Each covariate is listed with the source for the covariate and our justification for including this covariate in our covariate selection process.

Covariate	Source	Reasoning for testing
Completeness of NVSS	Global Burden of Disease[10]	Standard metrics related to the quality of vital statistics reporting
Percent garbage coding in NVSS		
Percent well-certified of NVSS		
Drug overdose mortality rate	Global Burden of Disease[17]	Causes of death commonly investigated by medical examiners and coroners
Suicide mortality rate		
Homicide mortality rate		
Lag distributed income (LDI) per capita by U.S. state	Global Burden of Disease[39,40]	Feldman et al found that county income quintile was correlated with underreporting of police violence in NVSS[41]
Lag distributed income (LDI) per capita by U.S. state, race, and ethnicity		
Lag distributed income (LDI) per capita by U.S. state for Non-Hispanic Black people		
Percent of police violence deaths in NVSS through the mechanism of firearms	NVSS	Feldman et al found that firearm vs non-firearm mechanism was correlated with underreporting of police violence in NVSS[41]
State	N/A	Death investigation systems are legislated and managed differently within each state, leading to different outcomes in data collection and reporting
Race/ethnicity group	N/A	Longstanding, documented systemic racism in the USA criminal justice system

Author contributions

Managing the estimation or publications process

Eve E Wool, Nicole Davis Weaver, Gregory A Roth, and Mohsen Naghavi.

Writing the first draft of the manuscript

Fablina Sharara, Eve E Wool, Nicole Davis Weaver, and Laurie B Marczak.

Primary responsibility for applying analytical methods to produce estimates

Fablina Sharara, Gregory J Bertolacci, Shelly Balassyano, Ilse N Dippenaar, Matthew Cunningham, Catherine Owens Johnson, and Mohsen Naghavi.

Primary responsibility for seeking, cataloguing, extracting, or cleaning data; designing or coding figures and tables

Fablina Sharara, Gregory J Bertolacci, Shelly Balassyano, Alexandrea Watson, John E Fuller, and Mohsen Naghavi. Fablina Sharara, Gregory J Bertolacci, Shelly Balassyano, Alexandrea Watson, John E Fuller, and Mohsen Naghavi.

Providing data or critical feedback on data sources

Gregory J Bertolacci, Shelly Balassyano, Gregory A Roth, Shailesh M Advani, Fares Alahdab, Sanjay Basu, Dejana Braithwaite, Samath Dhamminda Dharmaratne, Laura Dwyer-Lindgren, Islam Y Elgendy, Jawad Fares, Ali Gholamrezanezhad, Ayman Grada, Avirup Guha, Nima Hafezi-Nejad, Kevin S Ikuta, Khaled Khatab, Jagdish Khubchandani, Adnan Kisa, Vijay Krishnamoorthy, Kris J Krohn, Kate E LeGrand, Xuefeng Liu, Ted R Miller, Ali H Mokdad, Christopher J L Murray, Shrikant Pawar, Nicholas L S Roberts, David C Schwebel, Jasvinder A Singh, Bryan L Sykes, Ismaeel Yunusa, and Mohsen Naghavi.

Developing methods or computational machinery

Fablina Sharara, Eve E Wool, Gregory J Bertolacci, Shelly Balassyano, Ilse N Dippenaar, Matthew Cunningham, Gregory A Roth, Adnan Kisa, Ali H Mokdad, Christopher J L Murray, Robert C Reiner Jr, Nicholas L S Roberts, and Mohsen Naghavi.

Providing critical feedback on methods or results

Fablina Sharara, Eve E Wool, Gregory J Bertolacci, Nicole Davis Weaver, Shelly Balassyano, Matthew Cunningham, Aidin Abedi, Juan Manuel Acuna, Shailesh M Advani, Pradyumna Agasthi, Fares Alahdab, Ziyad Al-Aly, Shervin Assari, Asma Tahir Awan, Sanjay Basu, Arielle Wilder Bell, Dejana Braithwaite, Katrin Burkart, Daniel Youngwhan Cho, Mohiuddin Ahsanul Kabir Chowdhury, Xiaochen Dai, Farah Daoud, Laura Dwyer-Lindgren, Islam Y Elgendy, Jawad Fares, Medhat Farwati, James L Fisher, Ali Gholamrezanezhad, Sameer Vali Gopalani, Ayman Grada, Avirup Guha, Nima Hafezi-Nejad, Mohammad Rifat Haider, Simon I Hay, Md Mahbub Hossain, Kevin S Ikuta, Jessica Y Islam, Roxana Jabbarinejad, Khaled Khatab, Jagdish Khubchandani, Daniel Kim, Ruth W Kimokoti, Adnan Kisa, Vijay Krishnamoorthy, Kris J Krohn, Wayne R Lawrence, Kate E LeGrand, Stephen S Lim, Ziqiang Lin, Xuefeng Liu, Morteza Mahmoudi, Ramon Martinez-Piedra, Kala M Mehta, Ted R Miller, Modhurima Moitra, Ali H Mokdad, Christopher J L Murray, Anna María Nápoles, Elaine Okanyene Nsoesie, Shrikant Pawar, Pradhum Ram, Nicholas L S Roberts, Sana Salehi, Juan Sanabria, David C Schwebel, Inga Dora Sigfusdottir, Jasvinder A Singh, Bryan L Sykes, Hooman Tadbiri, Imad I Tleyjeh, Alexander C Tsai, Theo Vos, Chenkai Wu, Ismaeel Yunusa, Wangjian Zhang, and Mohsen Naghavi

Drafting the work or revising is critically for important intellectual content

Eve E Wool, Gregory J Bertolacci, Nicole Davis Weaver, Shelly Balassyano, Laurie B Marczak, Gregory A Roth, Juan Manuel Acuna, Shailesh M Advani, Pradyumna Agasthi, Fares Alahdab, Catherine M Antony, Shervin Assari, Asma Tahir Awan, Sanjay Basu, Arielle Wilder Bell, Dejana Braithwaite, Adrienne Chew,

Daniel Youngwhan Cho, Samath Dhamminda Dharmaratne, Laura Dwyer-Lindgren, Islam Y Elgendy, Jawad Fares, Medhat Farwati, James L Fisher, Sameer Vali Gopalani, Ayman Grada, Avirup Guha, Nima Hafezi-Nejad, Simon I Hay, Md Mahbub Hossain, Jessica Y Islam, Khaled Khatab, Jagdish Khubchandani, Adnan Kisa, Kris J Krohn, Kate E LeGrand, Kala M Mehta, Ted R Miller, Ali H Mokdad, Shane Douglas Morrison, Christopher J L Murray, Anna María Nápoles, Shrikant Pawar, Tessa M Pilz, Pradhum Ram, Nicholas L S Roberts, Sana Salehi, Juan Sanabria, David C Schwebel, Aziz Sheikh, Jasvinder A Singh, Bryan L Sykes, Hooman Tadbiri, Imad I Tleyjeh, Alexander C Tsai, Theo Vos, Srikanth Yandrapalli, Ramin Zand, and Mohsen Naghavi.

Extracting, cleaning, or cataloging data; designing or coding figures and tables
Gregory J Bertolacci, Shelly Balassyano, Alexandrea Watson, Matthew Cunningham, John E Fuller, Ali H Mokdad, Shrikant Pawar, Nicholas L S Roberts, Emma Elizabeth Spurlock, and Mohsen Naghavi.

Managing the overall research enterprise
Eve E Wool, Laurie B Marczak, Simon I Hay, Ali H Mokdad, Christopher J L Murray, Christopher M Odell, Emma Elizabeth Spurlock, and Mohsen Naghavi.
